# Distributed quantile regression for big data with missing covariates

**DOI:** 10.1007/s12561-026-09528-6

**Published:** 2026-07-09

**Authors:** Ye Fan, Hua Zhou, Jin Zhou, Tiejun Tong, Nan Lin

**Affiliations:** 1 School of Statistics and Data Science, Capital University of Economics and Business, Beijing, 100070 China; 2 Department of Computational Medicine, University of California, Los Angeles, CA 90095 USA; 3 Department of Biostatistics, University of California, Los Angeles, CA 90095 USA; 4 Department of Epidemiology and Biostatistics, University of Arizona, Tucson, AZ 85721 USA; 5 Department of Mathematics, Hong Kong Baptist University, Hong Kong, 999077 China; 6 Department of Statistics and Data Science, Washington University in St. Louis, St. Louis, MO 63130 USA

**Keywords:** Big data, Distributed algorithm, Missing covariates, Weighted quantile regression

## Abstract

Data missing caused by non-response or drop-out appears routinely in modern medical studies. With the rapid development of information technology, medical data are becoming more and more massive in volume, and thus usually require distributed analysis in real-world applications, especially for cases where the data are collected from multiple centers or contain patient privacy. In this paper, we focus on developing efficient distributed algorithms to support quantile regression analysis in missing big data, which provides a powerful tool to model the skew and heterogeneous medical samples in reality, but currently remains a challenging issue. We employ the weighted quantile regression (WQR) technique to incorporate the missing information into the model and propose two two-stage distributed algorithms, IPW-ADMM and IPW-renewable, for efficient and privacy-preserving estimation of WQR. Both methods first estimate the missingness mechanism using a logistic model and then solve the WQR problem in a communication-efficient manner without sharing raw individual-level data. The IPW-ADMM algorithm parallelizes estimation using a multi-block alternating direction method of multipliers (ADMM), reformulating the nonsmooth WQR objective into a set of local subproblems. The IPW-renewable algorithm adopts a sequential renewable estimation framework with the smoothing technique, making it suitable for streaming or incremental data settings. Simulation studies demonstrate that both proposed methods achieve estimation accuracy comparable to the classical centralized interior point (IP) method, while offering substantial computational speedups in distributed environments. An application to the UK Biobank dataset further illustrates their practical utility in detecting heterogeneous genetic associations across quantiles under covariate missingness.

**Supplementary Information:**

The online version contains supplementary material available at https://doi.org/10.1007/s12561-026-09528-6.

## Introduction

Modern biomedical studies now routinely involve large-scale datasets collected across multiple clinical centers, cohort studies, and biobank initiatives (e.g., UK Biobank). Although outcomes are typically well recorded, covariate information is often partially incomplete due to selective non-response on specific questionnaire items, variability in clinical data entry, or differences in data collection protocols across sites. In large observational databases, these patterns of covariate missingness can meaningfully affect estimation efficiency and validity if ignored [1]. Simply discarding subjects with incomplete covariates is inefficient and may distort downstream inference, making the appropriate incorporation of partially observed covariate information a critical challenge in contemporary biomedical analysis.

At the same time, the massive size and distributed storage of modern biomedical data create substantial computational barriers. When datasets are partitioned across institutions or computing nodes due to privacy, governance, or logistical constraints, standard estimation strategies are often difficult to deploy at scale. Biomedical outcomes also frequently display skewness and heterogeneous effect patterns, motivating the use of quantile regression (QR) [2] in applications ranging from epidemiology to genome-wide biobank studies [3]. In linear QR, for observations $\{(y_i, \boldsymbol{x}_i)\}_{i=1}^n$, the τth (0<τ<1) conditional quantile of $y_i\in \mathbb {R}$ is modeled as $Q_{y_i \mid \boldsymbol{x}_i}(\tau ) = \boldsymbol{x}_i^{\textrm{T}}\boldsymbol{\beta }(\tau )$, and the estimator of $\boldsymbol{\beta }\left( \tau \right)$ is obtained by solving

1 $$\begin{aligned} \widehat{\boldsymbol{\beta }}(\tau ) = \arg \min _{\boldsymbol{\beta }} \sum _{i=1}^n \rho _{\tau }\!\left( y_i - \boldsymbol{x}_i^{\textrm{T}}\boldsymbol{\beta }\right) , \end{aligned}$$

where $\pmb {x}_i\in \mathbb {R}^p$ and $\rho _{\tau }\left( \cdot \right)$ is the check loss function. When covariates are partially missing, direct application of this estimator discards incomplete observations and may lead to substantial efficiency loss or bias. A common remedy is to incorporate partially observed units through inverse probability weighting (IPW) [1], yielding a weighted quantile regression (WQR) formulation. However, the introduction of observation-specific weights makes the loss function nonseparable across samples, posing significant obstacles for distributed computation. State-of-the-art distributed QR algorithms [4–8] are designed for fully observed covariates and do not extend naturally to this weighted setting, leaving an important methodological gap in large-scale biomedical data analysis.

We note that our goal is to develop distributed computational strategies for the WQR framework, rather than to introduce a new identification strategy for missing data. Accordingly, the statistical validity of the target WQR estimator continues to rely on the usual assumptions underlying IPW, including missing at random (MAR) and correct specification of the missingness model. Under these conditions, the resulting weighted estimator retains its standard large-sample interpretation, while our contribution is to make its computation scalable in distributed settings. If the missingness model is misspecified, the distributed algorithms still solve their intended weighted optimization or updating problems conditional on the estimated weights, but the statistical target induced by those weights may be affected.

The novelty of our framework also goes beyond simply incorporating missing covariates into an existing distributed QR routine. Under IPW, both the responses and covariates are rescaled by the estimated missingness probabilities, which changes the optimization structure and may affect numerical behavior. Moreover, the missingness probabilities themselves must be estimated in a distributed manner. Therefore, existing divide-and-conquer or consensus-based QR methods are not directly transferable to this setting through a naive plug-in modification. This motivates the newly proposed procedures, which are designed to jointly handle distributed missingness model estimation and distributed computation for the WQR objective.

To address these challenges, we develop two complementary distributed algorithms for WQR with incomplete covariates. Both approaches follow a two-stage strategy: the missingness mechanism is first modeled based on fully observed quantities, after which the resulting inverse probability weights are incorporated into a distributed optimization framework for the WQR objective. The first method, IPW-ADMM, employs a multi-block alternating direction method of multipliers (ADMM) formulation that enables parallel updating of local QR parameters together with consensus enforcement across computing nodes. The second method, IPW-renewable, adapts the renewable estimation principle to update the missingness model and QR coefficients sequentially, allowing efficient site-by-site or streaming data computation without retaining historical data.

To our knowledge, these developments provide the first distributed frameworks capable of accommodating inverse probability WQR, a setting to which existing scalable QR algorithms do not extend. By requiring only low-dimensional summary information to be transmitted across sites, the proposed approaches can support privacy-preserving computation at biobank scale while remaining computationally efficient. Moreover, both methods achieve substantial speedups relative to centralized estimators while maintaining good statistical accuracy, as demonstrated through simulations and an analysis of UK Biobank data. These contributions fill an important methodological gap in the analysis of large, multisite biomedical datasets with incomplete covariate information.

The rest of this paper is organized as follows. In Sects. 2 and 3, we respectively give the algorithm details of the proposed IPW-ADMM and IPW-renewable for QR estimation with missing data. Section 4 contains simulation studies that evaluate the empirical performance of the proposed algorithms. In Sect. 5, we further analyze a UK Biobank data set to illustrate the practical performance of these two algorithms. Section 6 gives the concluding remarks.

## The IPW-ADMM algorithm

Let’s first briefly review the IPW-based WQR estimator [1] for the centralized case. We consider situations where the response $y_i$ is fully observed and the covariate vector $\boldsymbol{x}_i = (\boldsymbol{x}_i^{f}, \boldsymbol{x}_i^{m})$ consists of a fully observed part $\boldsymbol{x}_i^{f} \in \mathbb {R}^d$ and a partially observed part $\boldsymbol{x}_i^{m} \in \mathbb {R}^{p-d}$. Let $R_i$ indicate whether $\boldsymbol{x}_i^{m}$ is observed, and $\pi (\boldsymbol{z}_i, \pmb \gamma ) = P(R_i = 1 \mid \boldsymbol{z}_i)$ denote a model for the probability of missingness indexed by $\pmb \gamma$ based on fully observed variables $\boldsymbol{z}_i = (y_i, \boldsymbol{x}_i^{f})$. The WQR estimator is then defined as

$$\widehat{\boldsymbol{\beta }}^{\,w}(\tau ) = \arg \min _{\boldsymbol{\beta }} \sum _{i \in I} \frac{1}{\pi (\boldsymbol{z}_i,\widehat{\pmb \gamma })}\, \rho _{\tau }\!\left( y_i - \boldsymbol{x}_i^{\textrm{T}}\boldsymbol{\beta }\right) ,$$

where $I = \{i: R_i = 1\}$ and $\widehat{\pmb \gamma }\in \mathbb {R}^q$ is the estimated parameter in the missingness model. The formulation preserves the robustness of QR while adjusting for the informative nature of the missingness mechanism. Although conceptually straightforward, WQR introduces new computational difficulties: the observation-specific weights destroy the separability of the objective function across samples, which renders the optimization problem substantially more challenging in distributed environments.

Now consider a distributed setting in which the samples are stored across *M* local machines. The *m*th (m=1,2,⋯,M) machine contains observations $\{(y_{mi},\boldsymbol{x}_{mi})\}_{i = 1}^{n_m}$ with $\sum _{m=1}^{M} n_m = n$. Let $I_m = \{ i: R_{mi} = 1 \}$ denote the indices of units on machine *m* with fully observed covariates and let $n_m^{*} = |I_m|$. Under this notation, the WQR objective can be expressed in distributed form as

2 $$\begin{aligned} \widehat{\boldsymbol{\beta }}^{\,w}\left( \tau \right) = \arg \min _{\boldsymbol{\beta }} \sum _{m=1}^{M} \sum _{i \in I_m} \frac{1}{\pi (\boldsymbol{z}_{mi},\widehat{\pmb \gamma })}\, \rho _{\tau }\!\left( y_{mi} - \boldsymbol{x}_{mi}^{\textrm{T}}\boldsymbol{\beta }\right) . \end{aligned}$$

We next introduce a two-stage distributed method IPW-ADMM for computing $\widehat{\pmb \gamma }$ and solving (2). The first stage aggregates site-specific score and Hessian contributions to estimate the missingness model, and the second stage employs a multi-block ADMM formulation to optimize the WQR objective across machines while enforcing global consensus.

### Distributed computation for the weights

To compute the inverse probability weights $\pi (\boldsymbol{z}_{mi},\widehat{\pmb \gamma })$ in a distributed environment, we first estimate the parameter $\pmb \gamma$ in the missingness model. For simplicity, we assume a logistic regression model $\pi (\boldsymbol{z}_i, \pmb \gamma ) = P(R_i = 1 \mid \boldsymbol{z}_i)=\frac{\exp (\boldsymbol{z}_i^{\textrm{T}}\pmb \gamma )}{1 + \exp (\boldsymbol{z}_i^{\textrm{T}} \pmb \gamma )}$. In centralized computation, the parameter $\pmb \gamma$ is typically estimated via the Newton–Raphson algorithm, which updates

3 $$\begin{aligned} \boldsymbol{\gamma }^{k+1} = \boldsymbol{\gamma }^{k} - F_n^{-1}\!\left( \boldsymbol{\gamma }^{k}\right) S_n\!\left( \boldsymbol{\gamma }^{k}\right) , \end{aligned}$$

where

4 $$\begin{aligned} S_n(\boldsymbol{\gamma }^{k}) = \sum _{i=1}^{n} \boldsymbol{z}_i \!\left[ R_i - \frac{\exp (\boldsymbol{z}_i^{\textrm{T}}\boldsymbol{\gamma }^{k})}{1 + \exp (\boldsymbol{z}_i^{\textrm{T}}\boldsymbol{\gamma }^{k})} \right] \end{aligned}$$

and

5 $$\begin{aligned} F_n(\boldsymbol{\gamma }^{k}) = \sum _{i=1}^{n} \boldsymbol{z}_i\, \frac{\exp (\boldsymbol{z}_i^{\textrm{T}}\boldsymbol{\gamma }^{k})}{\left[ 1+\exp (\boldsymbol{z}_i^{\textrm{T}}\boldsymbol{\gamma }^{k})\right] ^{2}} \boldsymbol{z}_i^{\textrm{T}} \end{aligned}$$

denote the score vector and Hessian matrix, respectively.

Both the score and Hessian enjoy additive decompositions over data partitions,

6 $$\begin{aligned} S_n(\boldsymbol{\gamma }^{k}) = \sum _{m=1}^{M} S_{n_m}(\boldsymbol{\gamma }^{k}), \qquad F_n(\boldsymbol{\gamma }^{k}) = \sum _{m=1}^{M} F_{n_m}(\boldsymbol{\gamma }^{k}), \end{aligned}$$

where $S_{n_m}(\cdot )$ and $F_{n_m}(\cdot )$ are the contributions computed using only the observations stored on machine *m*. This additive structure makes the Newton–Raphson update naturally amenable to distributed implementation.

In each iteration, every local machine computes its own score and Hessian contributions, $S_{n_m}(\boldsymbol{\gamma }^k)$ and $F_{n_m}(\boldsymbol{\gamma }^k)$, and transmits them to a central coordinator. The coordinator aggregates these quantities according to (6) and updates $\boldsymbol{\gamma }^{k+1}$ using (3). Once convergence is reached, the weight for each observed unit is obtained as $\pi (\boldsymbol{z}_{mi},\widehat{\pmb \gamma })$, which depends only on the local covariates $\boldsymbol{z}_{mi}$. This completes the first stage of IPW-ADMM.

#### Remark 1

For very large-scale problems where forming Hessian contributions is computationally expensive or memory intensive, one may instead employ stochastic-gradient or incremental-gradient methods to estimate $\pmb \gamma$ [9, 10]. The IPW-ADMM framework does not rely on any specific optimizer for the missingness model, and such alternatives can be incorporated when needed.

#### Remark 2

The distributed computation in this stage concerns only how the missingness model estimator is obtained without sharing raw data. The statistical validity of the resulting inverse probability weights still depends on the usual modeling assumptions for IPW, including MAR and correct specification of the missingness model. If the model is misspecified, the distributed procedure still computes the corresponding estimated weights correctly, but the induced weighted estimator may no longer target the same population quantity as under correct specification.

### Distributed computation for the QR parameter

After obtaining the estimated weights $\pi (\boldsymbol{z}_{mi},\widehat{\pmb \gamma })$, we re-express the weighted samples on the *m*th machine as $(\boldsymbol{y}_m^{*}, X_m^{*})$, where $\boldsymbol{y}_m^{*} = (y_{m1}^{*},\ldots ,y_{m n_m^{*}}^{*})^{\textrm{T}}$ and $X_m^{*} = (\boldsymbol{x}_{m1}^{*},\ldots ,\boldsymbol{x}_{m n_m^{*}}^{*})^{\textrm{T}},$ with

$$y_{mi}^{*} = \pi ^{-1}(\boldsymbol{z}_{mi},\widehat{\pmb \gamma })\, y_{mi} \quad \text{ and } \quad \boldsymbol{x}_{mi}^{*} = \pi ^{-1}(\boldsymbol{z}_{mi},\widehat{\pmb \gamma })\, \boldsymbol{x}_{mi}.$$

As in standard IPW methods, the numerical impact of reweighting is most pronounced when some missingness probabilities are close to zero, which yields large weights. This issue is mitigated under the common positivity-type condition that there exists a constant $c_\pi>0$ such that $\pi (\boldsymbol{z},\pmb \gamma )\ge c_\pi$ for all $\boldsymbol{z}$.

Using the nonnegativity of the weights and the piecewise linearity of the check loss function, the WQR problem (2) can be written compactly as

7 $$\begin{aligned} \widehat{\boldsymbol{\beta }}^{\,w}\left( \tau \right) = \arg \min _{\boldsymbol{\beta }} \sum _{m=1}^{M} \rho _{\tau }\!\left( \boldsymbol{y}_m^{*} - X_m^{*}\boldsymbol{\beta }\right) . \end{aligned}$$

Here we write $\sum _{i \in I_m} \rho _{\tau }\left( y_{mi}^*-\pmb x_{mi}^{*\textrm{T}}\pmb \beta \right)$ as $\rho _{\tau }\left( \pmb y_m^*-X_m^*\pmb \beta \right)$ for notational simplicity.

To solve (7) distributively, we employ an ADMM approach. ADMM has become a widely used optimization framework in large-scale statistical and machine learning problems because it naturally decomposes global objectives into separable subproblems that can be solved in parallel across distributed computing nodes. Existing ADMM formulations for QR [7, 8, 11] typically rely on introducing a single residual-type auxiliary variable and applying the classical two-block ADMM. Such schemes solve an ordinary least squares subproblem in each iteration, which becomes unstable or infeasible when the design matrix is strongly correlated or high dimensional. In contrast, our IPW-ADMM algorithm is based on a multi-block ADMM formulation, which allows blockwise separation of local parameters and residuals across the *M* sites and avoids the restrictive least squares update. We first provide a brief review of the multi-block ADMM framework before describing its application to (7).

#### A brief review of multi-block ADMM

The classical ADMM [12, 13] is designed for solving linearly constrained optimization problems with two separable objective components and has been widely applied in large-scale regression and machine learning problems. The multi-block ADMM [14] extends it to problems involving more than two components. Specifically, it considers

8 $$\begin{aligned} \min _{\boldsymbol{z}_1,\ldots ,\boldsymbol{z}_L} \ \sum _{l=1}^L f_l(\boldsymbol{z}_l) \quad \text {s.t.}\quad \sum _{l=1}^L A_l \boldsymbol{z}_l = \boldsymbol{b}, \end{aligned}$$

where each block variable $\boldsymbol{z}_l \in \mathbb {R}^{p_l}$ is associated with a separable function $f_l(\cdot )$, the matrices $A_l \in \mathbb {R}^{n \times p_l}$ encode the linear constraint structure, and $\boldsymbol{b} \in \mathbb {R}^n$ is fixed.

Let $\mathcal {L}_{\eta }$ denote the augmented Lagrangian,

$$\mathcal {L}_{\eta }(\boldsymbol{z}_1,\ldots ,\boldsymbol{z}_L,\boldsymbol{u}) = \sum _{l=1}^L f_l(\boldsymbol{z}_l) + \boldsymbol{u}^{\textrm{T}}\! \left( \sum _{l=1}^L A_l \boldsymbol{z}_l - \boldsymbol{b}\right) + \frac{\eta }{2} \left\| \sum _{l=1}^L A_l \boldsymbol{z}_l - \boldsymbol{b}\right\| _2^2,$$

where $\boldsymbol{u}\in \mathbb {R}^n$ is the dual variable and η>0 is the penalty parameter. A typical multi-block ADMM iteration updates each block sequentially, followed by a dual ascent step:

9 $$\begin{aligned} \boldsymbol{z}_l^{k+1}&= \arg \min _{\boldsymbol{z}_l}\ \mathcal {L}_{\eta }\!\left( \boldsymbol{z}_1^{k+1}, \ldots , \boldsymbol{z}_{l-1}^{k+1}, \boldsymbol{z}_l, \boldsymbol{z}_{l+1}^{k}, \ldots , \boldsymbol{z}_L^{k}, \boldsymbol{u}^{k} \right) ,\qquad l=1,\ldots ,L, \nonumber \\ \boldsymbol{u}^{k+1}&= \boldsymbol{u}^k + \eta \left( \sum _{l=1}^L A_l \boldsymbol{z}_l^{k+1} - \boldsymbol{b} \right) . \end{aligned}$$

Because the objective $\sum _{l=1}^L f_l(\boldsymbol{z}_l)$ is separable, each subproblem in (9) involves only one block variable and is typically much simpler than the original problem (8). Equally important, these blockwise updates can often be carried out in parallel across multiple computing nodes, making multi-block ADMM especially attractive for large-scale or distributed statistical estimation problems. This ability to separate computation across data partitions is one of the main reasons ADMM has become a standard tool for distributed optimization.

#### ADMM formulation for distributed WQR

To apply the multi-block ADMM to WQR, we introduce local parameter copies $\boldsymbol{\beta }_m$ and local residual variables $\boldsymbol{r}_m = \boldsymbol{y}_m^{*} - X_m^{*}\boldsymbol{\beta }_m$, one for each site m=1,…,M. Problem (7) then becomes

10 $$\begin{aligned} \min _{\boldsymbol{\beta },\{\boldsymbol{\beta }_m\},\{\boldsymbol{r}_m\}} \ \sum _{m=1}^{M} \rho _{\tau }(\boldsymbol{r}_m) \quad \text {s.t.}\ \boldsymbol{\beta }_m = \boldsymbol{\beta },\; \boldsymbol{r}_m = \boldsymbol{y}_m^{*} - X_m^{*}\boldsymbol{\beta }_m, \; m=1,\ldots ,M. \end{aligned}$$

This structure fits naturally into the multi-block ADMM framework. Let $\pmb u_m \in \mathbb {R}^p$ and $\pmb v_m\in \mathbb {R}^{n_m^*}$ denote the dual variables associated with the constraints $\boldsymbol{\beta }_m=\boldsymbol{\beta }$ and $\boldsymbol{r}_m=\boldsymbol{y}_m^{*}-X_m^{*}\boldsymbol{\beta }_m$, respectively. Optimizing the augmented Lagrangian for (10) then leads to the following multi-block ADMM updates.

11 $$\begin{aligned} & \pmb \beta ^{k+1} = \arg \underset{\pmb \beta }{\min }\ \frac{\eta }{2}\sum _{m=1}^M\left\| \pmb \beta _m^k-\pmb \beta +\pmb u_m^k/\eta \right\| _2^2,\end{aligned}$$

12 $$\begin{aligned} & \pmb r_m^{k+1} = \arg \underset{\pmb r_m}{\min }\ \rho _{\tau }\left( \pmb r_m\right) +\frac{\eta }{2}\left\| \pmb y_m^*-\pmb X_m^* \pmb \beta _m^k-\pmb r_m+\pmb v_m^k/\eta \right\| _2^2,\end{aligned}$$

13 $$\begin{aligned} & \pmb \beta _m^{k+1} = \arg \underset{\pmb \beta _m}{\min }\ \frac{\eta }{2}\left( \left\| \pmb \beta _m-\pmb \beta ^{k+1}+\pmb u_m^k/\eta \right\| _2^2+\left\| \pmb y_m^*-\pmb X_m^*\pmb \beta _m-\pmb r_m^{k+1}+\pmb v_m^k/\eta \right\| _2^2\right) ,\end{aligned}$$

14 $$\begin{aligned} & \pmb u_m^{k+1}=\pmb u_m^k+\eta \left( \pmb \beta _m^{k+1}-\pmb \beta ^{k+1}\right) , \end{aligned}$$

15 $$\begin{aligned} & \pmb v_m^{k+1} = \pmb v_m^k +\eta \left( \pmb y_m^*-\pmb X_m^*\pmb \beta _m^{k+1}-\pmb r_m^{k+1} \right) , \end{aligned}$$

where η>0 is the augmentation parameter. Through introducing the additional auxiliary variables $\pmb \beta _m$’s, the local update for the regression parameter is inverted into a nonzero-centered ridge regression problem, i.e., (13), which regresses $\left( \pmb y_m^*-\pmb r_m^{k+1}+\pmb v_m^k/\eta \right)$ on $\pmb X_m^*$, and thus scales well to strongly correlated and/or high dimensional cases, for example, the cases when $\pmb X_m^{*\textrm{T}}\pmb X_m^{*}$ is non-invertible. This stabilization is particularly relevant after IPW, since the transformed design matrix becomes $X_m^*=W_mX_m$ with Gram matrix $X_m^{\textrm{T}}W_m^{\textrm{T}}W_mX_m$, where $W_m$ is the weighting matrix, so heterogeneous weights may worsen numerical conditioning. More importantly, all the subproblems in the algorithm have closed-form solutions can be computed in parallel across the *M* machines.

Update of the global parameter $\boldsymbol{\beta }$ in (11) can be solved by $\boldsymbol{\beta }^{k+1} = \bar{\boldsymbol{\beta }}^{\,k} + \bar{\boldsymbol{u}}^{\,k}/\eta$, where $\bar{\boldsymbol{\beta }}^{\,k} = \frac{1}{M}\sum _{m=1}^{M}\boldsymbol{\beta }_m^{k}$ and $\bar{\boldsymbol{u}}^{\,k} = \frac{1}{M}\sum _{m=1}^{M}\boldsymbol{u}_m^{k}.$

In (12), because $\rho _{\tau }\left( \cdot \right)$ is separable and piecewise linear, the optimization with respect to $\boldsymbol{r}_m$ decomposes into independent one-dimensional problems for each component of $\boldsymbol{r}_m$. Therefore, the update of the local residuals $\boldsymbol{r}_m$ at each machine *m* is obtained by applying the soft-thresholding rule elementwise, namely

$$\boldsymbol{r}_m^{k+1} = \left[ \boldsymbol{w}_m^{k} - \tau \boldsymbol{1}/\eta \right] _{+} - \left[ -\boldsymbol{w}_m^{k} + (1-\tau )\boldsymbol{1}/\eta \right] _{+},$$

where $\boldsymbol{w}_m^{k} = \boldsymbol{y}_m^{*} - X_m^{*}\boldsymbol{\beta }_m^{k} + \boldsymbol{v}_m^{k}/\eta$. That is, for each coordinate *j*,

$$r_{m,j}^{k+1} = \left[ w_{m,j}^{k} - \tau /\eta \right] _{+} - \left[ -w_{m,j}^{k} + (1-\tau )/\eta \right] _{+}.$$

In (13), update of the local parameters $\boldsymbol{\beta }_m$ at each site *m* is a nonzero-centered ridge regression problem, whose closed-form solution is

$$\boldsymbol{\beta }_m^{k+1} = \left( I_p + X_m^{*\textrm{T}} X_m^{*}\right) ^{-1} \Big [ \boldsymbol{\beta }^{k+1} - \boldsymbol{u}_m^{k}/\eta + X_m^{*\textrm{T}} ( \boldsymbol{y}_m^{*} - \boldsymbol{r}_m^{k+1} + \boldsymbol{v}_m^{k}/\eta ) \Big ].$$

Each iteration of this multi-block ADMM algorithm requires transmitting only the local pairs $(\boldsymbol{\beta }_m^{k}, \boldsymbol{u}_m^{k})$ to the central coordinator, making the algorithm communication-efficient and fully privacy-preserving. Further, it is worth noting that the matrices $(I_p + X_m^{*\textrm{T}} X_m^{*})$ are fixed across iterations, so they can be pre-inverted once per site. As a result, even under the centralized setting, the multi-block ADMM formulation can be computationally more efficient than the classic interior point method for QR, which repeatedly invert weighted design matrices. The entire algorithm is summarized in Algorithm 1.

**Algorithm 1:**
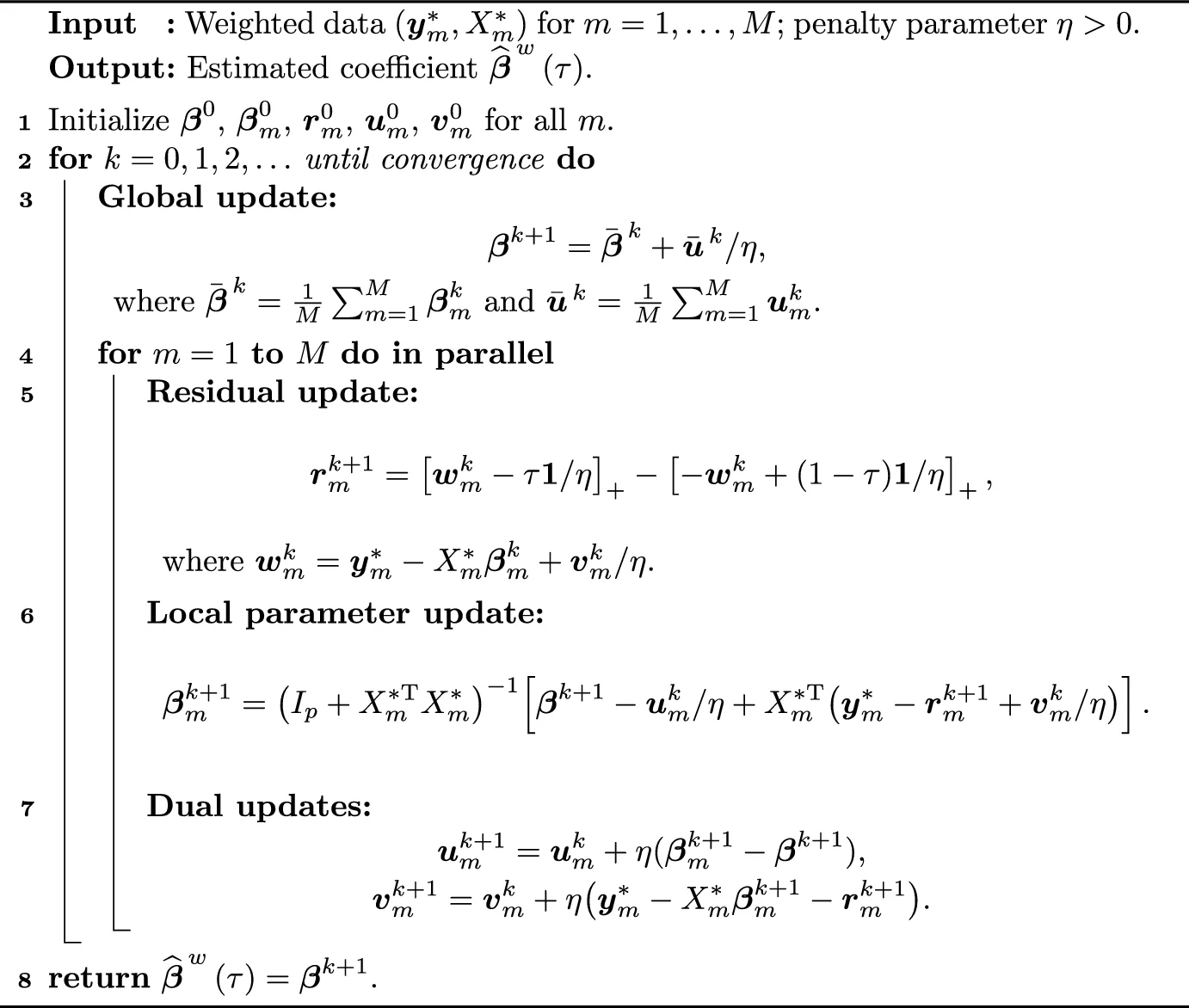
IPW-ADMM for distributed weighted quantile regression

#### The convergence theory of IPW-ADMM

The convergence of classic two-block ADMM for convex optimization problems has been well established in the literature [15]. For multi-block ADMM, although its convergence is not guaranteed in general, [14] pointed out that when two coefficient matrices in the linear constraint are orthogonal, the convergence of three-block ADMM can be established by applying similar proof techniques in [15]. For our problem (10), through a simple rearrangement of the linear constraints, we find that the coefficient matrices for variables $\boldsymbol{\beta }$ and $\left( {\pmb r}_1^{\textrm{T}},{\pmb r}_2^{\textrm{T}},\cdots , {\pmb r}_M^{\textrm{T}}\right) ^{\textrm{T}} \in \mathbb {R}^{n}$ respectively are $A_1 = \left( I_p, I_p,\cdots ,I_p, \pmb 0_{p\times n}\right) ^{\textrm{T}} \in \mathbb {R}^{\left( M\times p+n\right) \times p}$ and $A_2=\left( \pmb 0_{n\times \left( M\times p\right) }, I_n\right) ^{\textrm{T}}\in \mathbb {R}^{\left( M\times p+n\right) \times n}$, and satisfy $A_1^{\textrm{T}} A_2 = \pmb 0_{p\times n}.$ Therefore, the convergence of the IPW-ADMM is guaranteed as stated in the following theorem.

##### Theorem 1

Assume that the unaugmented Lagrangian function for (10), i.e.,

$$\mathcal {L}_{0}\left( \boldsymbol{\beta }, \pmb r_m, \boldsymbol{\beta }_m, \pmb u_m, \pmb v_m\right) =\sum _{m=1}^M \rho _{\tau }\left( \pmb r_m\right) +\sum _{m=1}^M {\pmb u}_m^{\textrm{T}} \left( \boldsymbol{\beta }_m-\boldsymbol{\beta }\right) +\sum _{m=1}^M {\pmb v}_m^{\textrm{T}} \left( \pmb y^{*}_m-X_m^{*} \boldsymbol{\beta }_m-\pmb r_m\right) ,$$

has a saddle point $\left( \boldsymbol{\beta }^*,\pmb r_m^*,\boldsymbol{\beta }_m^*,\pmb u_m^*,\pmb v_m^*\right) .$ Then the sequence $\left\{ \sum _{m=1}^M \rho _{\tau }\left( \pmb r_m^k\right) \right\}$ generated by IPW-ADMM converges to the minimum of (10). In addition, $\left( \boldsymbol{\beta }^k,\pmb r_m^k,\boldsymbol{\beta }_m^k\right)$ converges to an optimal solution to (10).

##### Proof

The detailed proofs are given in Sect. 2 in the Supplementary Materials. □

##### Remark 3

Assuming the existence of a saddle point is a standard assumption for the convergence of ADMM [15, 16], which implies that an optimal solution to (10) exists.

### Communication cost and local memory of IPW-ADMM

Recall $\boldsymbol{\beta }\in \mathbb {R}^p$ and $\boldsymbol{\gamma }\in \mathbb {R}^{d+1}$. The communication cost of IPW-ADMM arises from two stages. In the first stage, distributed logistic regression is used to estimate $\hat{\boldsymbol{\gamma }}$. At each Newton step, each site transmits its local score vector and Hessian contribution, whose sizes are (d+1) and (d+1)×(d+1), respectively. Thus, the communication size per site per Newton step is $\left[ d+1+(d+1)^2\right]$ entries.

In the second stage, the WQR problem is solved by ADMM. At each ADMM iteration, each site updates its local variables and transmits only low-dimensional parameter vectors needed for consensus enforcement. Hence, the communication size per site per ADMM iteration is of order *p*, independent of the local sample size $n_m$. Therefore, the QR-stage communication cost of IPW-ADMM is low-dimensional but iterative.

In terms of local memory, each site stores its local weighted data together with the precomputed matrix $\left( I_p+X_m^{*\mathrm T}X_m^*\right) ^{-1}$ used in the $\boldsymbol{\beta }_m$ update. Since the size of this symmetric matrix p×p is independent of the sample size, the additional algorithm-specific memory burden is moderate in the regime considered in this paper.

## The IPW-renewable algorithm

The IPW-ADMM algorithm introduced in Sect. 2 is well suited for distributed settings in which data stored across multiple machines can be processed through iterative global synchronization. In such batch-distributed environments, consensus-based optimization provides an effective way to update local and global parameters simultaneously while maintaining data locality and communication efficiency. In other situations, however, data may arrive sequentially or may need to be incorporated incrementally over time. Examples include large-scale biomedical studies that continually expand recruitment, collaborative projects in which different machines upload data asynchronously, or longitudinal monitoring systems that collect high-frequency signals from wearable or mobile health devices. In these contexts, new observations accumulate over time and model updates must often be incorporated without revisiting previous raw data, so an estimation strategy that supports sequential updating is more natural and computationally appropriate. We can easily connect this situation to the setup in Sect. 2 if data are observed in blocks and the block index *m* corresponds to the temporal order of data arrival.

To accommodate such scenarios, we develop a complementary estimation method, IPW-renewable, which updates the missingness parameter $\boldsymbol{\gamma }$ and the QR parameter $\boldsymbol{\beta }$ in a blockwise and sequential fashion. The approach is motivated by the broader renewable estimation framework for streaming and distributed data [17], which constructs global estimators by aggregating low-dimensional summary statistics rather than relying on iterative local estimators. The separation of the $\boldsymbol{\gamma }$- and $\boldsymbol{\beta }$-updates enables the method to operate efficiently in both streaming and distributed environments.

### Update of the weight parameter $\boldsymbol{\gamma }$

For simplicity, same as in Sect. 2.1, we model the missingness probability using a logistic regression, so that estimate of $\boldsymbol{\gamma }$ is defined as the solution to the global score equation

$$S_{n}(\boldsymbol{\gamma }) = \sum _{m=1}^{M} S_{n_m}(\boldsymbol{\gamma }) = \boldsymbol{0}_{d+1},$$

where $S_{n_m}(\boldsymbol{\gamma })$ is the local score based on data stored on machine *m*. Directly solving this equation in a distributed environment may be prohibitively restrictive due to storage and privacy constraints. The renewable estimation approach resolves this difficulty by updating $\boldsymbol{\gamma }$ sequentially across machines using only low-dimensional summary quantities rather than raw data.

Suppose the data on machines $1,\ldots ,(m\!-\!1)$ have already been incorporated, yielding the current estimate $\widehat{\boldsymbol{\gamma }}^{(m-1)}$. If we were to compute the full global estimate using all data on the *m* machines in a centralized setting, it would satisfy $\sum _{j=1}^{m} S_{n_j}(\boldsymbol{\gamma }) = \boldsymbol{0}_{d+1}.$ Note that we must avoid evaluating the accumulated score $\sum _{j=1}^{m-1} S_{n_j}(\boldsymbol{\gamma })$ on machine *m* because it requires transmitting data from machines 1,…,(m-1) to machine *m*. Instead, we consider its first order approximate around $\widehat{\boldsymbol{\gamma }}^{(m-1)}$,

$$\sum _{j=1}^{m-1} S_{n_j}(\boldsymbol{\gamma }) \approx -\Bigg [\sum _{j=1}^{m-1} F_{n_j}\!\left( \widehat{\boldsymbol{\gamma }}^{(m-1)}\right) \Bigg ] \big (\boldsymbol{\gamma } - \widehat{\boldsymbol{\gamma }}^{(m-1)}\big ),$$

where $F_{n_j}(\cdot )$ is the local Hessian. However, evaluating $F_{n_j}\!\left( \widehat{\boldsymbol{\gamma }}^{(m-1)}\right)$ on machine *m* still requires access to either the raw data from machine *j* or its Hessian contributions recomputed at the new parameter value $\widehat{\boldsymbol{\gamma }}^{(m-1)}$, neither of which is feasible under the distributed setting due to privacy and storage constraints. Following the renewable estimation strategy in [17], we further replace the Hessian matrix $F_{n_j}\!\left( \widehat{\boldsymbol{\gamma }}^{(m-1)}\right)$ with its evaluated value at the time machine *j* was processed, i.e., $F_{n_j}\!\left( \widehat{\boldsymbol{\gamma }}^{(j)}\right)$. This yields the renewable estimating equation:

16 $$\begin{aligned} S_{n_m}(\boldsymbol{\gamma }) - \Bigg [\sum _{j=1}^{m-1} F_{n_j}\!\left( \widehat{\boldsymbol{\gamma }}^{(j)}\right) \Bigg ] \big (\boldsymbol{\gamma } - \widehat{\boldsymbol{\gamma }}^{(m-1)}\big ) = \boldsymbol{0}_{d+1}, \end{aligned}$$

whose solution defines the updated estimate $\widehat{\boldsymbol{\gamma }}^{(m)}$.

In this implementation, machine *m* only requires two quantities from the previous step: (1) the accumulated Hessian matrix $\sum _{j=1}^{m-1} F_{n_j}\!\left( \widehat{\boldsymbol{\gamma }}^{(j)}\right)$ and (2) the current global estimate $\widehat{\boldsymbol{\gamma }}^{(m-1)}$. No raw data from earlier machines are transmitted, and the update can be performed using only the local data on machine *m*.

### Update of the QR parameter $\boldsymbol{\beta }$

Once the estimate $\widehat{\boldsymbol{\gamma }}$ has been obtained and the weights $\pi (\boldsymbol{z}_{mi},\widehat{\boldsymbol{\gamma }})$ have been computed, estimation of the QR parameter $\boldsymbol{\beta }$ reduces to solving the WQR problem in (7). In the IPW-renewable algorithm, $\boldsymbol{\beta }$ is updated across machines in a sequential manner that mirrors the renewable update for the missingness parameter in Sect. 3.1. However, the score based on the nonsmooth check loss,

$$S_{n_j^{*}}^{\textrm{QR}}(\boldsymbol{\beta }) = \sum _{i=1}^{n_j^{*}} \big [\mathbb {I}(y_{ji}^{*} - \boldsymbol{x}_{ji}^{*\textrm{T}}\boldsymbol{\beta } < 0) - \tau \big ] \boldsymbol{x}_{ji}^{*},$$

is discontinuous in $\boldsymbol{\beta }$ and does not admit a stable Taylor expansion. To apply the renewable strategy, we follow [18] and approximate $S_{n_j^{*}}^{\textrm{QR}}(\boldsymbol{\beta })$ by a smoothed score function $Q_{n_j^{*}}(\boldsymbol{\beta })$, obtained by convolving the indicator function with a kernel. The smoothed Hessian $\nabla Q_{n_j^{*}}(\boldsymbol{\beta })$ then plays the role analogous to the local Hessian $F_{n_j}\left( \cdot \right)$ in the renewable update for $\boldsymbol{\gamma }$.

Let $\widehat{\boldsymbol{\beta }}^{(m-1)}$ denote the current global estimate after machines 1,⋯,(m-1) have been processed. Applying the same renewable approximation as in Sect. 3.1, but using the smoothed score and Hessian, yields the estimating equation

17 $$\begin{aligned} Q_{n_m^{*}}(\boldsymbol{\beta }) + \Bigg [ \sum _{j=1}^{m-1} \nabla Q_{n_j^{*}}\!\left( \widehat{\boldsymbol{\beta }}^{(j)}\right) \Bigg ] \big (\boldsymbol{\beta } - \widehat{\boldsymbol{\beta }}^{(m-1)}\big ) = \boldsymbol{0}_p, \end{aligned}$$

whose solution defines the updated estimator $\widehat{\boldsymbol{\beta }}^{(m)}$. This formulation is directly analogous to (16), with the smoothed score $Q_{n_j^{*}}\left( \cdot \right)$ replacing the logistic regression score $S_{n_j}\left( \cdot \right)$ and the smoothed Hessian $\nabla Q_{n_j^{*}}\left( \cdot \right)$ replacing the logistic Hessian $F_{n_j}\left( \cdot \right)$.

Following [18], we solve (17) on machine *m* by gradient descent with a Barzilai–Borwein stepsize [19], which offers fast convergence and requires only machine-level computations together with the accumulated summaries from earlier machines. The IPW-renewable algorithm is summarized in Algorithm 2.

**Algorithm 2:**
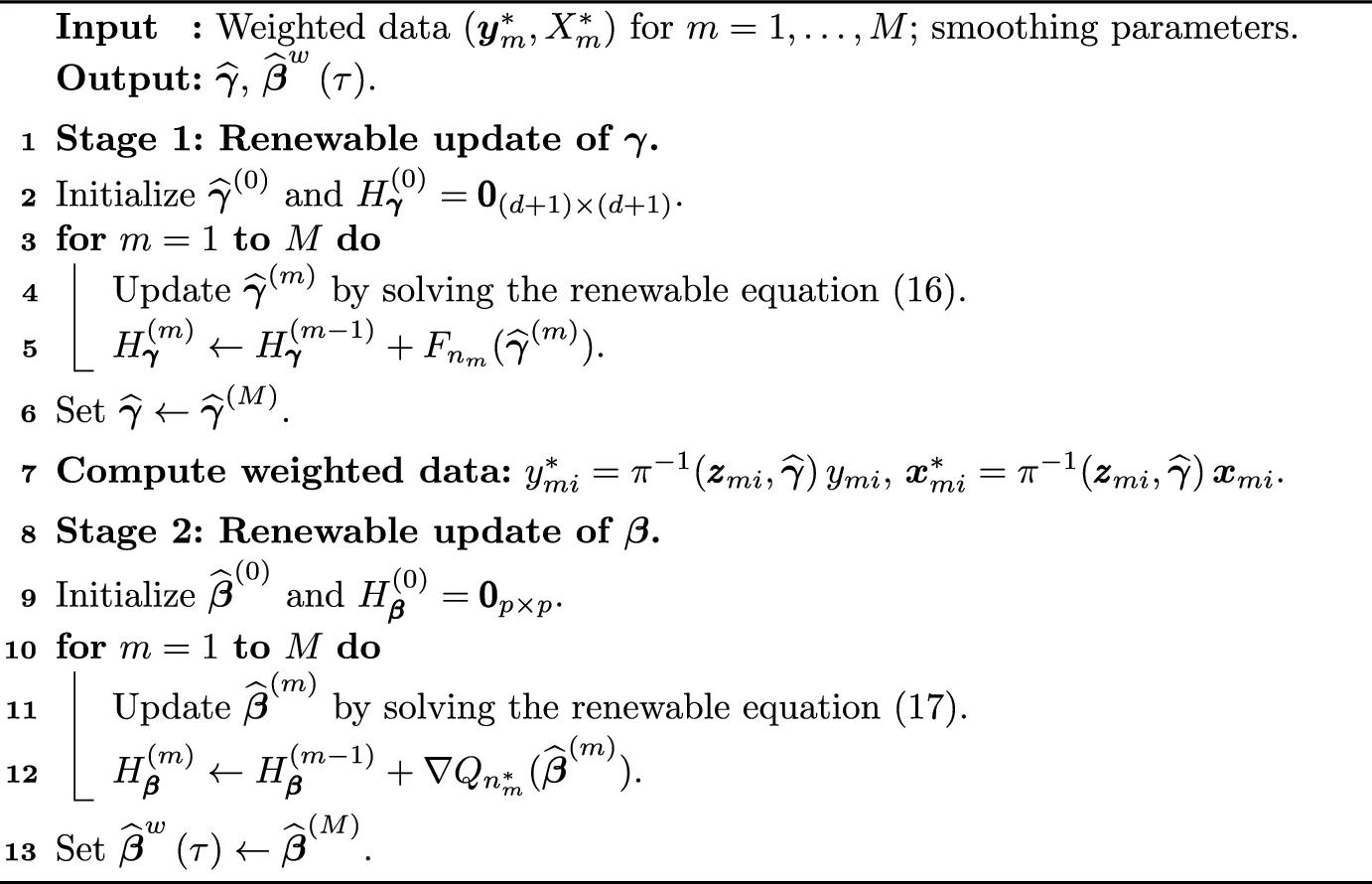
IPW-renewable for distributed weighted quantile regression

### Statistical property of the IPW-renewable estimator

In this section, we analyze the large-sample property of the IPW-renewable estimator. Let $Q\left( \boldsymbol{\beta }\right)$ be the population score of the QR outcome model, and $\boldsymbol{\beta }_0$ a solution to the equation $E\left[ Q\left( \boldsymbol{\beta }\right) \right] = \pmb 0_{p}$. Under mild regularity conditions, we can show that the IPW-renewable estimator $\hat{\boldsymbol{ \beta }}^{(M)}$ is consistent and asymptotically normal. Next, we define the regularity conditions in Assumption 1 and then give the theoretical property of IPW-renewable in Theorem 2.

**Assumption 1.**

- The parameter $\boldsymbol{\beta }_0$ is the unique solution to $E\left[ Q\left( \boldsymbol{\beta }\right) \right] = \pmb 0_{p}$;
- The kernel function used for smoothing in IPW-renewable is non-negative, bounded and Lipschitz continuous;
- The initial estimator $\widehat{\boldsymbol{\beta }}^{(0)}$ is asymptotically consistent.
- There exists a constant $c_{\pi }>0$ such that $\pi (\boldsymbol{z},\pmb \gamma ) \ge c_{\pi }$ for all $\boldsymbol{z}$ in the support of the fully observed variables.

Under Assumption 1, we have the following asymptotic property hold for the IPW-renewable estimator.

#### Theorem 2

The IPW-renewable estimator $\widehat{\boldsymbol{\beta }}^{(M)}$ is consistent and asymptotically normal. More specifically, we have $\hat{\boldsymbol{\beta }}^{(M)} \rightarrow _{p} \boldsymbol{\beta }_0$ and

$$\begin{aligned} \sqrt{n^{*}}\left( \widehat{\boldsymbol{\beta }}^{(M)}-\boldsymbol{\beta }_0\right) \rightarrow _d \mathcal {N}\left( \pmb 0_{p}, \left[ \nabla Q\left( \boldsymbol{\beta }_0\right) \right] ^{-1}E\left[ Q \left( \boldsymbol{\beta }_0\right) Q^{\textrm{T}}\left( \boldsymbol{\beta }_0\right) \right] \left[ \nabla Q\left( \boldsymbol{\beta }_0\right) \right] ^{-1}\right) , \end{aligned}$$

where $$n^{*} = \sum _{m=1}^M n_m^{*}$$.

The detailed proof of Theorem 2 is given in Sect. 3 in the Supplementary Materials. Using the same techniques, we can also prove the consistency and asymptotic normality of $\widehat{\boldsymbol{\gamma }}^{(M)}$.

The above asymptotic result is stated for the weighted estimating problem induced by the estimated missingness probabilities. As with standard IPW-based methods, its population interpretation relies on correct specification of the missingness model together with the MAR assumption. The theorem here characterizes the large-sample behavior of the renewable estimator relative to that weighted target; misspecification of the missingness model can alter the target itself.

In addition, the renewable update for the QR parameter relies on a smoothed score, which introduces an approximation bias for finite bandwidth. The main reason for smoothing is that the original quantile score is nonsmooth and therefore does not admit the type of Taylor expansion needed for renewable updating. By replacing it with a differentiable approximation, one can construct sequential score and Hessian-type updates using only low-dimensional summaries from previous blocks. At the same time, the bandwidth controls a bias–stability trade-off: if it is too large, the smoothed score deviates more from the original quantile score, increasing approximation bias; if it is too small, the smoothed problem becomes numerically less stable in finite samples. Under the bandwidth conditions assumed in Corollary 1 in [20], i.e., the bandwidth $h_m$ needs to satisfy $h_m\left( N_m/\log N_m\right) ^{1/3}\rightarrow \infty$ and $h_m = o\left( N_m^{-1/4}\right)$, where $N_m = \sum _{j = 1}^m n_j$ is the cumulative sample size, this smoothing bias is asymptotically negligible and therefore does not affect the consistency or asymptotic normality in Theorem 2. In our implementation, we use the theoretically motivated bandwidth rule $h_m=\left( N_m \log N_m\right) ^{-1/4}.$ Thus, in the streaming setting, the bandwidth is updated block by block as new data arrive. We also note that, although bandwidth selection is generally a nontrivial issue in smoothing problems, recent work on online smoothed QR suggests that estimation performance is relatively stable across a reasonable range of bandwidth values, so fine tuning is often not critical in practice.

### Communication cost comparison

Both IPW-ADMM and IPW-renewable avoid transmission of raw individual-level data and instead exchange only low-dimensional quantities, but they differ substantially in communication pattern. For IPW-ADMM, communication is iterative: after the first-stage distributed estimation of the missingness model, the weighted QR stage requires multiple ADMM communication rounds, with communication size per site per iteration of order *p*.

By contrast, IPW-renewable is sequential rather than iterative. In the renewable update of the missingness model parameter, the communicated summary consists of the current estimate and an accumulated Hessian-type matrix, with total size $\left[ d+1+(d+1)^2\right]$. In the renewable update of the weighted QR parameter, the communicated summary consists of the current estimate and an accumulated Hessian-type matrix for the smoothed QR step, with total size $\left( p+p^2\right)$. Thus, IPW-renewable requires larger message size per pass than IPW-ADMM, but it typically needs only one pass over the sites rather than repeated global communication rounds.

Compared with existing distributed QR methods, the main additional challenge in our setting is that the weighted QR objective is not fixed *a priori*; rather, it is induced by a first-stage distributed estimate of the missingness model. Therefore, both proposed methods have an intrinsic two-stage communication structure, first for obtaining the inverse probability weights and then for solving the induced weighted QR problem. Compared with IPW-ADMM, IPW-renewable has a simpler local memory structure. During processing at site *m*, both methods must of course hold the current site’s data, but IPW-ADMM additionally maintains ADMM-specific local variables and the precomputed p×p matrix $\left( I_p+X_m^{*\mathrm T}X_m^*\right) ^{-1}$ for repeated iterative updates. By contrast, IPW-renewable only requires the current site’s data together with the incoming estimator and propagated low-dimensional summaries.

## Simulation studies

This section evaluates the statistical and computational performance of the proposed IPW-ADMM and IPW-renewable algorithms. All simulations are implemented in R on a Mac computer equipped with an Apple M1 chip (8-core CPU, 8-core GPU, 16-core Neural Engine) and 16GB RAM. The source code of IPW-ADMM and IPW-renewable is available at https://github.com/yefan319/IPW.

### Setup

We generate synthetic data of size $(n,p) \in \{(50{,}000,50),\ (50{,}000,100),\ (200{,}000,100)\}$ from the linear model

18 $$\begin{aligned} y_i = \beta _0 + \sum _{j=1}^p x_{ij} \beta _j + \sigma _i \varepsilon _i, \end{aligned}$$

where the covariates are drawn independently with $x_{ij} \sim N(0,1)$ for j=1,…,0.8p and $x_{ij} \sim \textrm{Bernoulli}(0.5)$ for j=0.8p+1,…,p. That is the cumulative sample size $N_M = 50,000$ or 200, 000. The true coefficients are set to $\beta _0=-3$, $\beta _j = (-1)^{j-1}$ for j≤0.8p, and $\beta _j = 1$ for the remaining binary covariates. The independent error term satisfies $\varepsilon _i \sim N(0,1)$, and we consider both homoscedastic errors with $\sigma _i=5$ and heteroscedastic errors with $\sigma _i = 5 + x_{i1}$.

To mimic a MAR setting, we assume the covariates $(x_1,\ldots ,x_{0.4p},\; x_{0.8p+1},\ldots ,x_p)$ are fully observed, whereas $(x_{0.4p+1},\ldots ,x_{0.8p})$ are subject to missingness. The missing indicator *R* is generated from the logistic model

$$\textrm{logit}\!\left[ \Pr (R=1\mid y,x_1,\ldots ,x_5)\right] = 1 + 0.01\,y + 0.4\sum _{j=1}^5 x_j,$$

leading to an average missing rate of approximately 22%–25% across all settings.

### Results

We compute the WQR estimator at three quantile levels $\tau \in \{0.3, 0.5, 0.7\}$ using both IPW-ADMM and IPW-renewable. For comparison, we also include the oracle centralized benchmark estimator, denoted as IP. This method follows the same two-stage structure as our proposed approaches but with centralized estimation at both stages. The missingness model is fitted on the full dataset using the standard Newton–Raphson algorithm, and the resulting weights are then used to solve the weighted QR problem via the interior point (IP) method implemented in the rq() function of the quantreg package in R. Because it operates on the complete dataset without communication or storage constraints, the IP estimator serves as an oracle benchmark for evaluating estimation accuracy and computational efficiency.

We measure estimation accuracy by the absolute error $\textrm{AE} = \sum _{j=0}^p \big |\widehat{\beta }_j - \beta _j\big |.$ We also monitor their computational efficiency by recording $\textrm{T}_{\textrm{W}}$, the time for estimating the weights (i.e., solving for $\widehat{\boldsymbol{\gamma }}$), and $\textrm{T}_{\textrm{WQR}}$, the time for solving the WQR problem. The total computational time is then $\textrm{T}_{\textrm{total}} = \textrm{T}_{\textrm{W}} + \textrm{T}_{\textrm{WQR}}$.

Tables 1, 2, 3 report the results under homoscedastic errors for τ=0.3,0.5,0.7, respectively, and Tables 4, 5,  6 report the corresponding results under heteroscedastic errors. Across Tables 1, 2, 3, 4, 5, and 6, *M* denotes the number of data partitions. All reported values are averages over 100 Monte Carlo replicates, with standard deviations shown in parentheses.

**Table 1:** AE and computation time for IPW-ADMM, IPW-renewable, and the centralized IP estimator under homoscedastic error at τ=0.3

Method	*M*	AE	$\textrm{T}_{\textrm{total}}$ (s)	$\textrm{T}_{\textrm{W}}$ (s)	$\textrm{T}_{\textrm{WQR}}$ (s)
$$\left( n, p\right) =\left( 50,000, 50\right)$$					
IPW-ADMM	5	1.86(0.24)	0.19(0.04)	0.13(0.04)	0.06(0.01)
	10	1.89(0.25)	0.10(0.01)	0.06(0.01)	0.03(0.01)
	20	1.91(0.26)	0.06(0.02)	0.04(0.02)	0.02(0.01)
	50	1.91(0.24)	0.02(0.01)	0.01(0.01)	0.01(0.00)
	100	1.93(0.23)	0.02(0.02)	0.01(0.02)	0.01(0.00)
IPW-renewable	5	1.98(0.26)	0.68(0.03)	0.44(0.02)	0.24(0.02)
	10	2.08(0.33)	0.61(0.03)	0.38(0.02)	0.23(0.02)
	20	2.45(0.45)	0.56(0.03)	0.33(0.01)	0.23(0.02)
	50	2.61(0.84)	0.53(0.02)	0.30(0.02)	0.23(0.02)
	100	3.49(0.95)	0.50(0.02)	0.28(0.01)	0.22(0.02)
IP		1.87(0.23)	1.33(0.05)	0.65(0.05)	0.68(0.03)
$$\left( n, p\right) =\left( 50,000, 100\right)$$					
IPW-ADMM	5	3.62(0.35)	0.74(0.02)	0.49(0.01)	0.25(0.02)
	10	3.62(0.33)	0.42(0.03)	0.25(0.03)	0.17(0.02)
	20	3.67(0.33)	0.25(0.04)	0.13(0.04)	0.12(0.01)
	50	3.70(0.33)	0.13(0.02)	0.05(0.02)	0.08(0.01)
	100	3.71(0.32)	0.10(0.03)	0.03(0.03)	0.06(0.01)
IPW-renewable	5	3.83(0.39)	2.44(0.07)	1.62(0.03)	0.82(0.07)
	10	4.56(0.57)	2.12(0.06)	1.33(0.02)	0.78(0.05)
	20	4.95(0.65)	1.96(0.06)	1.19(0.02)	0.78(0.06)
	50	5.11(1.23)	1.83(0.07)	1.08(0.03)	0.76(0.06)
	100	6.68(1.45)	1.75(0.06)	1.01(0.02)	0.74(0.05)
IP		3.63(0.32)	4.12(0.18)	1.91(0.17)	2.21(0.09)
$$\left( n, p\right) =\left( 200,000, 100\right)$$					
IPW-ADMM	5	1.81(0.17)	2.68(0.20)	1.89(0.20)	0.79(0.02)
	10	1.82(0.17)	1.43(0.13)	1.02(0.13)	0.41(0.01)
	20	1.82(0.17)	0.70(0.08)	0.49(0.07)	0.22(0.01)
	50	1.82(0.15)	0.31(0.04)	0.21(0.04)	0.10(0.00)
	100	1.86(0.15)	0.17(0.03)	0.11(0.03)	0.06(0.00)
IPW-renewable	5	1.93(0.17)	9.84(0.25)	6.97(0.14)	2.87(0.19)
	10	2.06(0.22)	8.58(0.22)	5.81(0.10)	2.76(0.18)
	20	2.32(0.34)	7.56(0.17)	4.93(0.08)	2.63(0.14)
	50	2.49(0.52)	7.18(0.16)	4.57(0.10)	2.61(0.11)
	100	2.93(0.55)	6.98(0.16)	4.35(0.07)	2.63(0.15)
IP		1.81(0.16)	17.72(0.92)	7.95(0.85)	9.77(0.33)

**Table 2:** AE and computation time for IPW-ADMM, IPW-renewable, and the centralized IP estimator under homoscedastic error at τ=0.5

Method	*M*	AE	$\textrm{T}_{\textrm{total}}$ (s)	$\textrm{T}_{\textrm{W}}$ (s)	$\textrm{T}_{\textrm{WQR}}$ (s)
$$\left( n, p\right) =\left( 50,000, 50\right)$$					
IPW-ADMM	5	1.76(0.23)	0.19(0.04)	0.13(0.04)	0.06(0.01)
	10	1.80(0.22)	0.09(0.01)	0.06(0.01)	0.03(0.01)
	20	1.83(0.23)	0.05(0.02)	0.04(0.02)	0.01(0.01)
	50	1.86(0.24)	0.02(0.01)	0.01(0.01)	0.01(0.00)
	100	1.90(0.23)	0.02(0.02)	0.01(0.02)	0.01(0.00)
IPW-renewable	5	1.85(0.27)	0.66(0.03)	0.44(0.02)	0.22(0.02)
	10	1.97(0.31)	0.60(0.03)	0.38(0.02)	0.22(0.02)
	20	2.18(0.39)	0.56(0.02)	0.33(0.01)	0.22(0.02)
	50	2.37(0.69)	0.53(0.02)	0.30(0.02)	0.22(0.02)
	100	2.88(0.82)	0.49(0.02)	0.28(0.01)	0.21(0.01)
IP		1.76(0.23)	1.22(0.05)	0.65(0.05)	0.58(0.02)
$$\left( n, p\right) =\left( 50,000, 100\right)$$					
IPW-ADMM	5	3.35(0.30)	0.71(0.01)	0.49(0.01)	0.22(0.01)
	10	3.37(0.30)	0.40(0.03)	0.25(0.03)	0.15(0.02)
	20	3.39(0.31)	0.24(0.04)	0.13(0.04)	0.11(0.01)
	50	3.43(0.31)	0.12(0.02)	0.05(0.02)	0.07(0.01)
	100	3.48(0.34)	0.09(0.03)	0.03(0.03)	0.06(0.01)
IPW-renewable	5	3.49(0.38)	2.36(0.06)	1.62(0.03)	0.74(0.05)
	10	4.06(0.53)	2.08(0.06)	1.33(0.02)	0.75(0.05)
	20	4.21(0.60)	1.94(0.07)	1.19(0.02)	0.75(0.06)
	50	4.72(0.81)	1.83(0.07)	1.08(0.03)	0.76(0.06)
	100	5.77(1.28)	1.73(0.06)	1.01(0.02)	0.73(0.06)
IP		3.36(0.32)	3.86(0.21)	1.91(0.17)	1.94(0.09)
$$\left( n, p\right) =\left( 200,000, 100\right)$$					
IPW-ADMM	5	1.68(0.15)	2.59(0.20)	1.89(0.20)	0.70(0.01)
	10	1.68(0.15)	1.38(0.13)	1.02(0.13)	0.36(0.01)
	20	1.68(0.16)	0.68(0.07)	0.49(0.07)	0.19(0.01)
	50	1.68(0.14)	0.30(0.04)	0.21(0.04)	0.09(0.00)
	100	1.71(0.14)	0.16(0.03)	0.11(0.03)	0.05(0.00)
IPW-renewable	5	1.80(0.18)	9.60(0.22)	6.97(0.14)	2.63(0.14)
	10	1.98(0.21)	8.43(0.16)	5.81(0.10)	2.61(0.13)
	20	2.11(0.24)	7.43(0.15)	4.93(0.08)	2.50(0.12)
	50	2.36(0.34)	7.10(0.15)	4.57(0.10)	2.52(0.11)
	100	2.57(0.52)	6.90(0.14)	4.35(0.07)	2.55(0.11)
IP		1.68(0.15)	16.39(0.94)	7.95(0.85)	8.44(0.35)

**Table 3:** AE and computation time for IPW-ADMM, IPW-renewable, and the centralized IP estimator under homoscedastic error at τ=0.7

Method	*M*	AE	$\textrm{T}_{\textrm{total}}$ (s)	$\textrm{T}_{\textrm{W}}$ (s)	$\textrm{T}_{\textrm{WQR}}$ (s)
$$\left( n, p\right) =\left( 50,000, 50\right)$$					
IPW-ADMM	5	1.86(0.26)	0.19(0.12)	0.13(0.04)	0.06(0.04)
	10	1.88(0.28)	0.09(0.09)	0.06(0.01)	0.03(0.01)
	20	1.89(0.29)	0.06(0.09)	0.04(0.02)	0.02(0.02)
	50	1.94(0.29)	0.02(0.06)	0.01(0.01)	0.01(0.01)
	100	1.96(0.30)	0.02(0.04)	0.01(0.02)	0.01(0.02)
IPW-renewable	5	1.98(0.29)	0.68(0.03)	0.44(0.02)	0.24(0.02)
	10	2.02(0.31)	0.61(0.03)	0.38(0.02)	0.23(0.02)
	20	2.30(0.38)	0.56(0.02)	0.33(0.01)	0.22(0.01)
	50	2.61(0.67)	0.53(0.02)	0.30(0.02)	0.22(0.02)
	100	3.41(0.81)	0.50(0.02)	0.28(0.01)	0.21(0.01)
IP		1.86(0.25)	1.34(0.06)	0.65(0.05)	0.69(0.03)
$$\left( n, p\right) =\left( 50,000, 100\right)$$					
IPW-ADMM	5	3.35(0.30)	0.71(0.01)	0.49(0.01)	0.22(0.01)
	10	3.37(0.30)	0.40(0.03)	0.25(0.03)	0.15(0.02)
	20	3.39(0.31)	0.24(0.04)	0.13(0.04)	0.11(0.01)
	50	3.43(0.31)	0.12(0.02)	0.05(0.02)	0.07(0.01)
	100	3.48(0.34)	0.09(0.03)	0.03(0.03)	0.06(0.01)
IPW-renewable	5	3.86(0.37)	2.43(0.07)	1.62(0.03)	0.81(0.06)
	10	4.41(0.58)	2.11(0.06)	1.33(0.02)	0.77(0.06)
	20	4.71(0.63)	1.96(0.06)	1.19(0.02)	0.77(0.06)
	50	4.95(1.25)	1.83(0.06)	1.08(0.03)	0.76(0.05)
	100	6.47(1.51)	1.75(0.06)	1.01(0.02)	0.74(0.06)
IP		3.36(0.32)	3.86(0.21)	1.91(0.17)	1.94(0.09)
$$\left( n, p\right) =\left( 200,000, 100\right)$$					
IPW-ADMM	5	1.68(0.15)	2.59(0.20)	1.89(0.20)	0.70(0.01)
	10	1.68(0.15)	1.38(0.13)	1.02(0.13)	0.36(0.01)
	20	1.68(0.16)	0.68(0.07)	0.49(0.07)	0.19(0.01)
	50	1.68(0.14)	0.30(0.04)	0.21(0.04)	0.09(0.00)
	100	1.71(0.14)	0.16(0.03)	0.11(0.03)	0.05(0.00)
IPW-renewable	5	1.90(0.17)	9.84(0.20)	6.97(0.14)	2.87(0.16)
	10	2.12(0.21)	8.57(0.16)	5.81(0.10)	2.76(0.15)
	20	2.37(0.28)	7.55(0.15)	4.93(0.08)	2.62(0.12)
	50	2.58(0.47)	7.17(0.14)	4.57(0.10)	2.60(0.10)
	100	2.93(0.62)	6.96(0.15)	4.35(0.07)	2.61(0.13)
IP		1.68(0.15)	16.39(0.94)	7.95(0.85)	8.44(0.35)

**Table 4:** AE and computation time for IPW-ADMM, IPW-renewable, and the centralized IP estimator under heteroscedastic error at τ=0.3

Method	*M*	AE	$\textrm{T}_{\textrm{total}}$ (s)	$\textrm{T}_{\textrm{W}}$ (s)	$\textrm{T}_{\textrm{WQR}}$ (s)
$$\left( n, p\right) =\left( 50,000, 50\right)$$					
IPW-ADMM	5	1.81(0.21)	0.19(0.03)	0.13(0.03)	0.06(0.01)
	10	1.82(0.22)	0.09(0.02)	0.06(0.02)	0.03(0.01)
	20	1.86(0.24)	0.05(0.03)	0.03(0.03)	0.02(0.01)
	50	1.88(0.24)	0.03(0.01)	0.02(0.01)	0.01(0.00)
	100	1.92(0.23)	0.02(0.02)	0.01(0.02)	0.01(0.00)
IPW-renewable	5	1.92(0.28)	0.68(0.03)	0.44(0.01)	0.24(0.02)
	10	2.00(0.34)	0.61(0.02)	0.37(0.01)	0.23(0.02)
	20	2.35(0.45)	0.56(0.02)	0.33(0.01)	0.23(0.02)
	50	2.49(0.69)	0.53(0.03)	0.30(0.01)	0.23(0.02)
	100	3.15(0.92)	0.50(0.02)	0.28(0.01)	0.22(0.02)
IP		1.78(0.21)	1.31(0.05)	0.63(0.04)	0.68(0.03)
$$\left( n, p\right) =\left( 50,000, 100\right)$$					
IPW-ADMM	5	3.47(0.29)	0.72(0.02)	0.48(0.02)	0.24(0.01)
	10	3.47(0.29)	0.42(0.03)	0.25(0.02)	0.16(0.02)
	20	3.52(0.31)	0.25(0.02)	0.13(0.02)	0.12(0.01)
	50	3.55(0.30)	0.13(0.02)	0.05(0.02)	0.08(0.01)
	100	3.57(0.29)	0.09(0.02)	0.03(0.02)	0.06(0.01)
IPW-renewable	5	3.86(0.40)	2.45(0.08)	1.62(0.02)	0.83(0.07)
	10	4.22(0.48)	2.13(0.06)	1.33(0.02)	0.80(0.06)
	20	4.99(0.59)	1.98(0.07)	1.19(0.02)	0.79(0.06)
	50	5.55(0.99)	1.85(0.07)	1.08(0.01)	0.77(0.06)
	100	6.64(1.31)	1.76(0.06)	1.01(0.01)	0.75(0.05)
IP		3.47(0.30)	4.11(0.19)	1.90(0.17)	2.21(0.09)
$$\left( n, p\right) =\left( 200,000, 100\right)$$					
IPW-ADMM	5	1.73(0.16)	2.62(0.21)	1.85(0.21)	0.77(0.01)
	10	1.73(0.16)	1.37(0.12)	0.97(0.12)	0.40(0.01)
	20	1.73(0.15)	0.67(0.06)	0.46(0.06)	0.21(0.00)
	50	1.75(0.14)	0.29(0.03)	0.19(0.03)	0.10(0.00)
	100	1.78(0.15)	0.17(0.03)	0.11(0.03)	0.06(0.00)
IPW-renewable	5	1.84(0.18)	9.77(0.25)	6.90(0.13)	2.87(0.22)
	10	1.97(0.21)	8.58(0.18)	5.78(0.05)	2.79(0.18)
	20	2.29(0.29)	7.54(0.15)	4.89(0.04)	2.66(0.14)
	50	2.51(0.44)	7.16(0.13)	4.52(0.05)	2.64(0.13)
	100	3.24(0.55)	6.96(0.13)	4.33(0.04)	2.63(0.12)
IP		1.73(0.15)	17.56(0.84)	7.75(0.78)	9.81(0.34)

**Table 5:** AE and computation time for IPW-ADMM, IPW-renewable, and the centralized IP estimator under heteroscedastic error at τ=0.5

Method	*M*	AE	$\textrm{T}_{\textrm{total}}$ (s)	$\textrm{T}_{\textrm{W}}$ (s)	$\textrm{T}_{\textrm{WQR}}$ (s)
$$\left( n, p\right) =\left( 50,000, 50\right)$$					
IPW-ADMM	5	1.68(0.21)	0.19(0.03)	0.13(0.03)	0.05(0.01)
	10	1.74(0.22)	0.09(0.02)	0.06(0.02)	0.03(0.01)
	20	1.79(0.21)	0.05(0.03)	0.03(0.03)	0.01(0.01)
	50	1.81(0.22)	0.03(0.01)	0.02(0.01)	0.01(0.00)
	100	1.88(0.23)	0.02(0.02)	0.01(0.02)	0.01(0.00)
IPW-renewable	5	1.74(0.25)	0.67(0.03)	0.44(0.01)	0.23(0.02)
	10	1.87(0.27)	0.60(0.02)	0.37(0.01)	0.22(0.02)
	20	2.16(0.33)	0.56(0.07)	0.33(0.01)	0.23(0.07)
	50	2.31(0.61)	0.53(0.03)	0.30(0.01)	0.22(0.02)
	100	2.86(0.67)	0.49(0.02)	0.28(0.01)	0.21(0.02)
IP		1.69(0.21)	1.21(0.05)	0.63(0.04)	0.57(0.03)
$$\left( n, p\right) =\left( 50,000, 100\right)$$					
IPW-ADMM	5	3.22(0.26)	0.70(0.02)	0.48(0.02)	0.22(0.01)
	10	3.22(0.28)	0.39(0.03)	0.25(0.02)	0.14(0.02)
	20	3.25(0.30)	0.23(0.02)	0.13(0.02)	0.11(0.01)
	50	3.28(0.30)	0.12(0.02)	0.05(0.02)	0.07(0.01)
	100	3.31(0.30)	0.09(0.02)	0.03(0.02)	0.06(0.01)
IPW-renewable	5	3.40(0.41)	2.37(0.06)	1.62(0.02)	0.75(0.05)
	10	3.75(0.46)	2.09(0.06)	1.33(0.02)	0.76(0.06)
	20	4.29(0.68)	1.95(0.05)	1.19(0.02)	0.76(0.05)
	50	4.61(1.04)	1.84(0.06)	1.08(0.01)	0.76(0.06)
	100	6.16(1.28)	1.75(0.06)	1.01(0.01)	0.74(0.06)
IP		3.21(0.30)	3.85(0.19)	1.90(0.17)	1.94(0.08)
$$\left( n, p\right) =\left( 200,000, 100\right)$$					
IPW-ADMM	5	1.60(0.15)	2.54(0.21)	1.85(0.21)	0.69(0.01)
	10	1.61(0.14)	1.32(0.12)	0.97(0.12)	0.35(0.01)
	20	1.62(0.14)	0.65(0.06)	0.46(0.06)	0.18(0.00)
	50	1.60(0.14)	0.27(0.03)	0.19(0.03)	0.09(0.00)
	100	1.63(0.14)	0.16(0.03)	0.11(0.03)	0.05(0.00)
IPW-renewable	5	1.71(0.17)	9.59(0.21)	6.90(0.13)	2.68(0.17)
	10	1.90(0.19)	8.41(0.14)	5.78(0.05)	2.63(0.13)
	20	2.14(0.24)	7.39(0.11)	4.89(0.04)	2.50(0.10)
	50	2.35(0.31)	7.07(0.14)	4.52(0.05)	2.55(0.12)
	100	2.79(0.48)	6.93(0.14)	4.33(0.04)	2.60(0.13)
IP		1.61(0.15)	16.21(0.86)	7.75(0.78)	8.46(0.33)

**Table 6:** AE and computation time for IPW-ADMM, IPW-renewable, and the centralized IP estimator under heteroscedastic error at τ=0.7

Method	*M*	AE	$\textrm{T}_{\textrm{total}}$ (s)	$\textrm{T}_{\textrm{W}}$ (s)	$\textrm{T}_{\textrm{WQR}}$ (s)
$$\left( n, p\right) =\left( 50,000, 50\right)$$					
IPW-ADMM	5	1.85(0.24)	0.19(0.03)	0.13(0.03)	0.06(0.01)
	10	1.90(0.27)	0.09(0.02)	0.06(0.02)	0.03(0.01)
	20	1.94(0.26)	0.05(0.03)	0.03(0.03)	0.01(0.01)
	50	1.96(0.27)	0.03(0.01)	0.02(0.01)	0.01(0.00)
	100	1.98(0.28)	0.02(0.02)	0.01(0.02)	0.01(0.00)
IPW-renewable	5	1.98(0.28)	0.69(0.02)	0.44(0.01)	0.25(0.02)
	10	2.04(0.37)	0.61(0.02)	0.37(0.01)	0.23(0.02)
	20	2.37(0.42)	0.56(0.01)	0.33(0.01)	0.23(0.01)
	50	2.52(0.61)	0.53(0.02)	0.30(0.01)	0.23(0.02)
	100	3.05(0.93)	0.50(0.02)	0.28(0.01)	0.22(0.02)
IP		1.78(0.23)	1.32(0.05)	0.63(0.04)	0.69(0.04)
$$\left( n, p\right) =\left( 50,000, 100\right)$$					
IPW-ADMM	5	3.30(0.28)	0.71(0.02)	0.48(0.02)	0.23(0.01)
	10	3.31(0.28)	0.40(0.03)	0.25(0.02)	0.15(0.01)
	20	3.30(0.32)	0.24(0.02)	0.13(0.02)	0.11(0.01)
	50	3.33(0.29)	0.13(0.02)	0.05(0.02)	0.07(0.01)
	100	3.40(0.31)	0.09(0.02)	0.03(0.02)	0.06(0.01)
IPW-renewable	5	3.85(0.39)	2.43(0.05)	1.62(0.02)	0.81(0.05)
	10	4.14(0.50)	2.12(0.05)	1.33(0.02)	0.79(0.05)
	20	4.88(0.66)	1.99(0.14)	1.19(0.02)	0.80(0.14)
	50	5.35(0.99)	1.84(0.05)	1.08(0.01)	0.77(0.05)
	100	6.68(1.34)	1.76(0.06)	1.01(0.01)	0.75(0.06)
IP		3.28(0.28)	4.18(0.18)	1.90(0.17)	2.27(0.07)
$$\left( n, p\right) =\left( 200,000, 100\right)$$					
IPW-ADMM	5	1.64(0.15)	2.57(0.21)	1.85(0.21)	0.72(0.01)
	10	1.63(0.14)	1.34(0.12)	0.97(0.12)	0.37(0.01)
	20	1.65(0.15)	0.65(0.06)	0.46(0.06)	0.19(0.00)
	50	1.64(0.13)	0.28(0.03)	0.19(0.03)	0.09(0.00)
	100	1.67(0.15)	0.16(0.03)	0.11(0.03)	0.05(0.00)
IPW-renewable	5	1.82(0.16)	9.81(0.23)	6.90(0.13)	2.91(0.18)
	10	2.03(0.20)	8.61(0.19)	5.78(0.05)	2.83(0.18)
	20	2.17(0.25)	7.54(0.15)	4.89(0.04)	2.65(0.15)
	50	2.44(0.39)	7.15(0.15)	4.52(0.05)	2.63(0.13)
	100	3.17(0.56)	6.97(0.14)	4.33(0.04)	2.64(0.13)
IP		1.63(0.14)	18.11(0.81)	7.75(0.78)	10.36(0.35)

Overall, IPW-ADMM and IPW-renewable both achieve estimation accuracy that is broadly comparable to the centralized IP estimator across all (*n*, *p*) configurations, quantile levels, and error structures. As seen in Tables 1, 2, 3 (homoscedastic errors) and Tables 4, 5, 6 (heteroscedastic errors), the AE of IPW-ADMM is very close to that of the centralized IP solution for all values of *M*. This is expected, since IPW-ADMM optimizes exactly the same WQR objective as the centralized method (up to numerical tolerance), but in a distributed manner.

In contrast, IPW-renewable consistently exhibits somewhat larger AE, with the difference becoming more pronounced as *M* increases. Additional experiments suggest that this partition dependence is not driven by smoothing alone. In particular, the centralized smoothed *conquer* estimator [18] has estimation accuracy nearly identical to that of the centralized IP estimator across all reported settings; see Tables 7 and 8 ($(n, p) = (50{,}000, 50)$). This indicates that centralized smoothing itself does not introduce a substantial loss relative to the centralized benchmark. By contrast, the larger error of IPW-renewable arises mainly from the accumulation of approximation error in the renewable updating steps, including both the renewable update for the missingness model parameter and the renewable update for the WQR parameter, with the effect becoming more visible as the number of partitions increases.

**Table 7:** AE and computation time for IPW-renewable, the centralized IP and *conquer* estimator under homoscedastic error

Method	*M*	AE	$\textrm{T}_{\textrm{total}}$ (s)	$\textrm{T}_{\textrm{W}}$ (s)	$\textrm{T}_{\textrm{WQR}}$ (s)
τ=0.3					
IPW-renewable	5	1.98(0.26)	0.68(0.03)	0.44(0.02)	0.24(0.02)
	10	2.08(0.33)	0.61(0.03)	0.38(0.02)	0.23(0.02)
	20	2.45(0.45)	0.56(0.03)	0.33(0.01)	0.23(0.02)
	50	2.61(0.84)	0.53(0.02)	0.30(0.02)	0.23(0.02)
	100	3.49(0.95)	0.50(0.02)	0.28(0.01)	0.22(0.02)
Conquer		1.87(0.22)	0.89(0.05)	0.65(0.05)	0.24(0.02)
IP		1.87(0.23)	1.33(0.05)	0.65(0.05)	0.68(0.03)
τ=0.5					
IPW-renewable	5	1.85(0.27)	0.66(0.03)	0.44(0.02)	0.22(0.02)
	10	1.97(0.31)	0.60(0.03)	0.38(0.02)	0.22(0.02)
	20	2.18(0.39)	0.56(0.02)	0.33(0.01)	0.22(0.02)
	50	2.37(0.69)	0.53(0.02)	0.30(0.02)	0.22(0.02)
	100	2.88(0.82)	0.49(0.02)	0.28(0.01)	0.21(0.01)
Conquer		1.75(0.23)	0.82(0.05)	0.65(0.05)	0.17(0.02)
IP		1.76(0.23)	1.22(0.05)	0.65(0.05)	0.58(0.02)
τ=0.7					
IPW-renewable	5	1.98(0.29)	0.68(0.03)	0.44(0.02)	0.24(0.02)
	10	2.02(0.31)	0.61(0.03)	0.38(0.02)	0.23(0.02)
	20	2.30(0.38)	0.56(0.02)	0.33(0.01)	0.22(0.01)
	50	2.61(0.67)	0.53(0.02)	0.30(0.02)	0.22(0.02)
	100	3.41(0.81)	0.50(0.02)	0.28(0.01)	0.21(0.01)
Conquer		1.85(0.25)	0.90(0.05)	0.65(0.05)	0.25(0.03)
IP		1.86(0.25)	1.34(0.06)	0.65(0.05)	0.69(0.03)

**Table 8:** AE and computation time for IPW-renewable, the centralized IP and *conquer* estimator under heteroscedastic error

Method	*M*	AE	$\textrm{T}_{\textrm{total}}$ (s)	$\textrm{T}_{\textrm{W}}$ (s)	$\textrm{T}_{\textrm{WQR}}$ (s)
τ=0.3					
IPW-renewable	5	1.92(0.28)	0.68(0.03)	0.44(0.01)	0.24(0.02)
	10	2.00(0.34)	0.61(0.02)	0.37(0.01)	0.23(0.02)
	20	2.35(0.45)	0.56(0.02)	0.33(0.01)	0.23(0.02)
	50	2.49(0.69)	0.53(0.03)	0.30(0.01)	0.23(0.02)
	100	3.15(0.92)	0.50(0.02)	0.28(0.01)	0.22(0.02)
Conquer		1.77(0.21)	0.87(0.04)	0.63(0.04)	0.24(0.03)
IP		1.78(0.21)	1.31(0.05)	0.63(0.04)	0.68(0.03)
τ=0.5					
IPW-renewable	5	1.74(0.25)	0.67(0.03)	0.44(0.01)	0.23(0.02)
	10	1.87(0.27)	0.60(0.02)	0.37(0.01)	0.22(0.02)
	20	2.16(0.33)	0.56(0.07)	0.33(0.01)	0.23(0.07)
	50	2.31(0.61)	0.53(0.03)	0.30(0.01)	0.22(0.02)
	100	2.86(0.67)	0.49(0.02)	0.28(0.01)	0.21(0.02)
Conquer		1.67(0.21)	0.83(0.05)	0.65(0.05)	0.17(0.02)
IP		1.69(0.21)	1.21(0.05)	0.63(0.04)	0.57(0.03)
τ=0.7					
IPW-renewable	5	1.98(0.28)	0.69(0.02)	0.44(0.01)	0.25(0.02)
	10	2.04(0.37)	0.61(0.02)	0.37(0.01)	0.23(0.02)
	20	2.37(0.42)	0.56(0.01)	0.33(0.01)	0.23(0.01)
	50	2.52(0.61)	0.53(0.02)	0.30(0.01)	0.23(0.02)
	100	3.05(0.93)	0.50(0.02)	0.28(0.01)	0.22(0.02)
Conquer		1.77(0.23)	0.91(0.05)	0.65(0.05)	0.25(0.03)
IP		1.78(0.23)	1.32(0.05)	0.63(0.04)	0.69(0.04)

This effect is further amplified by the quality of the initial estimate. In our implementation, the renewable procedure is initialized using the estimator from the first data block. When the total sample size is fixed, increasing *M* reduces the size of that first block, making the starting estimator less accurate and allowing its error to propagate through subsequent renewable updates. To verify this effect, we conducted an additional experiment in which the initial estimate in IPW-renewable was replaced by a fixed value; see Table 9. Under this modification, the estimation accuracy improves substantially for larger *M*. These additional results suggest that the partition effect observed in our current simulations is driven largely by the weaker first-block-based initialization rather than by a fundamental instability of the renewable method itself. In practice, IPW-renewable is most attractive when communication or storage constraints make repeated global synchronization undesirable, when the number of partitions is moderate rather than extremely large, and when a reasonably accurate initial estimator is available. In broader applications, one may also have access to historical data, pilot data, or other external information that can be used to construct a stronger initial estimator, in which case this issue would be expected to be much less pronounced. We also note that our current results do not support a simple linear or sublinear accumulation rule in *M*; rather, the observed partition effect appears to reflect an interaction between renewable approximation and initialization quality. Thus, IPW-renewable remains a practically useful sequential alternative, especially when single-pass updating and limited storage are important.

**Table 9:** Impact of initial estimate on IPW-renewable (under homoscedastic error)

*M*	AE	$\textrm{T}_{\textrm{total}}$ (s)	$\textrm{T}_{\textrm{W}}$ (s)	$\textrm{T}_{\textrm{WQR}}$ (s)
τ=0.3				
IPW-renewable				
5	1.98(0.26)	0.68(0.03)	0.44(0.02)	0.24(0.02)
10	2.08(0.33)	0.61(0.03)	0.38(0.02)	0.23(0.02)
20	2.45(0.45)	0.56(0.03)	0.33(0.01)	0.23(0.02)
50	2.61(0.84)	0.53(0.02)	0.30(0.02)	0.23(0.02)
100	3.49(0.95)	0.50(0.02)	0.28(0.01)	0.22(0.02)
IPW-renewable with fixed initial estimate				
5	1.95(0.31)	0.68(0.03)	0.44(0.02)	0.24(0.02)
10	1.98(0.32)	0.61(0.03)	0.38(0.02)	0.23(0.02)
20	2.19(0.32)	0.59(0.04)	0.33(0.01)	0.26(0.03)
50	2.38(0.33)	0.57(0.04)	0.30(0.02)	0.26(0.02)
100	2.55(0.41)	0.54(0.04)	0.28(0.01)	0.26(0.03)
τ=0.5				
IPW-renewable				
5	1.85(0.27)	0.66(0.03)	0.44(0.02)	0.22(0.02)
10	1.97(0.31)	0.60(0.03)	0.38(0.02)	0.22(0.02)
20	2.18(0.39)	0.56(0.02)	0.33(0.01)	0.22(0.02)
50	2.37(0.69)	0.53(0.02)	0.30(0.02)	0.22(0.02)
100	2.88(0.82)	0.49(0.02)	0.28(0.01)	0.21(0.01)
IPW-renewable with fixed initial estimate				
5	1.81(0.26)	0.66(0.03)	0.44(0.02)	0.22(0.02)
10	1.91(0.27)	0.60(0.03)	0.38(0.02)	0.22(0.02)
20	2.03(0.28)	0.59(0.03)	0.33(0.01)	0.25(0.02)
50	2.13(0.29)	0.56(0.03)	0.30(0.02)	0.25(0.02)
100	2.32(0.30)	0.53(0.03)	0.28(0.01)	0.25(0.02)
τ=0.7				
IPW-renewable				
5	1.98(0.29)	0.68(0.03)	0.44(0.02)	0.24(0.02)
10	2.02(0.31)	0.61(0.03)	0.38(0.02)	0.23(0.02)
20	2.30(0.38)	0.56(0.02)	0.33(0.01)	0.22(0.01)
50	2.61(0.67)	0.53(0.02)	0.30(0.02)	0.22(0.02)
100	3.41(0.81)	0.50(0.02)	0.28(0.01)	0.21(0.01)
IPW-renewable with fixed initial estimate				
5	1.88(0.26)	0.68(0.03)	0.44(0.02)	0.24(0.02)
10	1.91(0.27)	0.61(0.03)	0.38(0.02)	0.23(0.02)
20	2.04(0.28)	0.58(0.03)	0.33(0.01)	0.25(0.02)
50	2.35(0.29)	0.58(0.02)	0.30(0.02)	0.28(0.02)
100	2.47(0.30)	0.52(0.03)	0.28(0.01)	0.26(0.03)

To further disentangle the roles of smoothing, renewable approximation, and initialization, we report additional diagnostic simulation results in Tables 7, 8, 9. Specifically, Tables 7 and 8 compare IPW-renewable with the centralized smoothed *conquer* estimator and the centralized IP estimator under the same settings as the main simulations, while Table 9 examines the effect of replacing the original first-block-based initial estimate by a fixed initial value.

Returning to the primary simulation results in Tables 1, 2, 3, 4, 5, 6 for IPW-ADMM, IPW-renewable, and IP, IPW-ADMM consistently delivers the smallest total time $\textrm{T}_{\textrm{total}}$ among the three methods. As *M* increases, $\textrm{T}_{\textrm{total}}$ for IPW-ADMM decreases markedly, reflecting the benefit of parallelization across machines in both weight estimation and WQR. In contrast, the centralized IP estimator via rq() is substantially slower, and its runtime grows rapidly with *n* and *p*.

IPW-renewable is implemented sequentially and therefore is slower than IPW-ADMM for a fixed (*n*, *p*, *M*), but it still offers a substantial reduction in computational cost compared with the centralized IP approach. Moreover, its sequential, summary-based structure makes it particularly suitable for settings where data arrive over time or must be incorporated without storing the entire history.

The simulation results show that IPW-ADMM preserves both the statistical and optimization properties of the centralized WQR estimator while providing significant computational gains in distributed batch settings. IPW-renewable offers a complementary alternative that trades a modest increase in estimation error for sequential, communication-efficient updates that are well suited to streaming or incremental data scenarios.

### Sensitivity to missingness model misspecification

To examine sensitivity to misspecification of the missingness model, we conducted an additional simulation study under the same setting as described above with $(n,p)=(50{,}000,50)$, but intentionally omitted the outcome *y* from the fitted logistic missingness model. This creates a mild misspecification relative to the data-generating mechanism, while keeping the rest of the setup unchanged. We considered the same quantile levels $\tau \in \{0.3,0.5,0.7\}$ under both homoscedastic and heteroscedastic errors.

Tables 10 reports the results. Several patterns are consistent across settings. First, the centralized IP benchmark itself shows some degradation in estimation accuracy under misspecification, as expected. Second, IPW-ADMM remains very close to the centralized IP estimator across all reported quantiles and partition sizes, indicating that the distributed ADMM implementation does not introduce noticeable additional sensitivity to this mild misspecification. Third, IPW-renewable shows the same qualitative pattern as in the correctly specified case: its estimation error is somewhat larger and tends to increase with the number of partitions, reflecting the additional approximation introduced by the renewable updating scheme rather than a distinct instability caused by distributed computation. Overall, these results suggest that the proposed distributed methods inherit the misspecification behavior of the underlying centralized WQR approach rather than amplifying it.

**Table 10:** AE and computation time for IPW-ADMM, IPW-renewable and the centralized IP estimator under missingness model misspecification (homoscedastic error)

Method	*M*	AE	$\textrm{T}_{\textrm{total}}$ (s)	$\textrm{T}_{\textrm{W}}$ (s)	$\textrm{T}_{\textrm{WQR}}$ (s)
τ=0.3					
IPW-ADMM	5	2.01(0.23)	0.20(0.04)	0.14(0.04)	0.06(0.01)
	10	2.08(0.24)	0.09(0.01)	0.06(0.01)	0.03(0.01)
	20	2.13(0.24)	0.05(0.02)	0.03(0.01)	0.02(0.01)
	50	2.17(0.25)	0.03(0.01)	0.02(0.02)	0.01(0.00)
	100	2.25(0.26)	0.02(0.02)	0.02(0.02)	0.01(0.00)
IPW-renewable	5	2.09(0.28)	0.67(0.03)	0.43(0.02)	0.24(0.02)
	10	2.26(0.33)	0.60(0.02)	0.37(0.02)	0.23(0.02)
	20	2.64(0.45)	0.56(0.02)	0.33(0.01)	0.23(0.02)
	50	3.10(0.80)	0.53(0.04)	0.30(0.02)	0.23(0.03)
	100	3.80(0.94)	0.50(0.03)	0.28(0.02)	0.22(0.02)
IP		1.89(0.22)	1.30(0.05)	0.61(0.03)	0.69(0.04)
τ=0.5					
IPW-ADMM	5	1.81(0.24)	0.20(0.04)	0.14(0.04)	0.06(0.01)
	10	1.89(0.24)	0.09(0.01)	0.06(0.01)	0.03(0.01)
	20	1.93(0.26)	0.05(0.01)	0.03(0.01)	0.01(0.01)
	50	1.96(0.24)	0.03(0.02)	0.02(0.02)	0.01(0.01)
	100	2.03(0.25)	0.02(0.02)	0.02(0.02)	0.01(0.01)
IPW-renewable	5	1.97(0.28)	0.65(0.03)	0.43(0.02)	0.23(0.02)
	10	2.19(0.34)	0.59(0.02)	0.37(0.02)	0.22(0.02)
	20	2.43(0.38)	0.55(0.03)	0.33(0.01)	0.22(0.02)
	50	2.75(0.71)	0.52(0.02)	0.30(0.02)	0.22(0.02)
	100	3.20(0.79)	0.49(0.02)	0.28(0.02)	0.21(0.02)
IP		1.80(0.23)	1.19(0.04)	0.61(0.03)	0.58(0.03)
τ=0.7					
IPW-ADMM	5	1.94(0.26)	0.20(0.04)	0.14(0.04)	0.06(0.01)
	10	2.01(0.29)	0.09(0.01)	0.06(0.01)	0.03(0.01)
	20	2.08(0.28)	0.05(0.01)	0.03(0.01)	0.02(0.01)
	50	2.08(0.30)	0.03(0.02)	0.02(0.02)	0.01(0.01)
	100	2.14(0.29)	0.02(0.02)	0.02(0.02)	0.01(0.01)
IPW-renewable	5	2.11(0.30)	0.67(0.03)	0.43(0.02)	0.24(0.02)
	10	2.34(0.36)	0.61(0.03)	0.37(0.02)	0.24(0.03)
	20	2.63(0.44)	0.56(0.02)	0.33(0.01)	0.23(0.01)
	50	3.16(0.77)	0.53(0.02)	0.30(0.02)	0.23(0.02)
	100	3.98(0.89)	0.49(0.02)	0.28(0.02)	0.21(0.01)
IP		1.90(0.23)	1.29(0.05)	0.61(0.03)	0.68(0.03)

## Real data analysis

In this section, we further apply the proposed IPW-ADMM and IPW-renewable algorithms to data from the UK Biobank, which provides a realistic large-scale setting with complex dependence structure and diverse covariate types for evaluating the performance of our proposed algorithms. We should note that the purpose of this section is methodological validation rather than a formal UK Biobank analysis.

Our analysis is based on the body mass index (BMI) measurements derived from the UKB primary care electronic health record (EHR) system. Following [21], clinical biomarker values in UKB are extracted from the gp_clinical table, which contains longitudinal primary care measurements encoded under Read v2 and CTV3 terminologies. The extracted cohort contains 144, 414 individuals (see Table 2 in [21]). For the purposes of this methodological comparison, we restrict the analysis to each individual’s first available BMI measurement.

Our QR model takes BMI as the response and includes the following covariates: age, age$^2$, sex, the age–sex interaction, the first ten genetic principal components (PCs), and a SNP of interest. Letting G denote the SNP genotype, the conditional quantile function at level τ is specified as

$$Q_{y \mid \pmb x}(\tau ) = \beta _0+ \beta _{\textrm{SNP}}\, G + \beta _1\, \text {age} + \beta _2\, \text {age}^2 + \beta _3\, \text {sex} + \beta _4\, (\text {age}\times \text {sex}) + \sum _{k=1}^{10} \gamma _k\, \textrm{PC}_k.$$

The PCs are computed from genome-wide genotype data following standard UKB procedures using high-quality, LD-pruned autosomal variants with long-range LD regions removed. Incorporating these PCs reduces confounding from subtle population substructure when estimating quantile-specific SNP effects.

Although UKB primary care measurements do contain real missing values, our starting dataset is fully observed after preprocessing (see details in [21]). To evaluate the proposed methods under a missing data setting while retaining a complete data reference for benchmarking, we introduce missingness as follows. Specifically, we treat the covariates

$$x_1=\text {age},\quad x_2=\text {age}^2,\quad x_3=\text {sex},\quad x_4=\text {age}\times \text {sex},\quad x_5=\text {PC}_1$$

as always observed, and impose missingness only on the remaining covariates. For each individual, missingness is generated from the logistic model

$$\textrm{logit}\{ P(R=1 \mid y, x_1,\ldots ,x_5) \} = 1 + 0.01\, y + \sum _{j=1}^5 0.01\, x_j,$$

where y denotes BMI. This design produces about 30% missingness. We note that the missingness in this experiment was introduced artificially so that the complete data analysis could be used as a benchmarking reference. In naturally occurring biobank missingness settings, the mechanism may be substantially more complex and may require additional modeling assumptions or surrogate-based strategies; see, for example [22, 23]. Therefore, the present analysis should be interpreted as a controlled illustration of method behavior rather than a complete treatment of realistic biobank missingness.

For illustration, we analyze the quantile-specific association between BMI and a well-established obesity-related SNP, rs17782313, located near the *MC4R* gene on chromosome 18. This locus has been repeatedly implicated in large-scale GWAS of BMI, making it a natural reference SNP for assessing whether the proposed distributed WQR methods recover heterogeneous genetic effects across the BMI distribution.

**Fig. 1:**
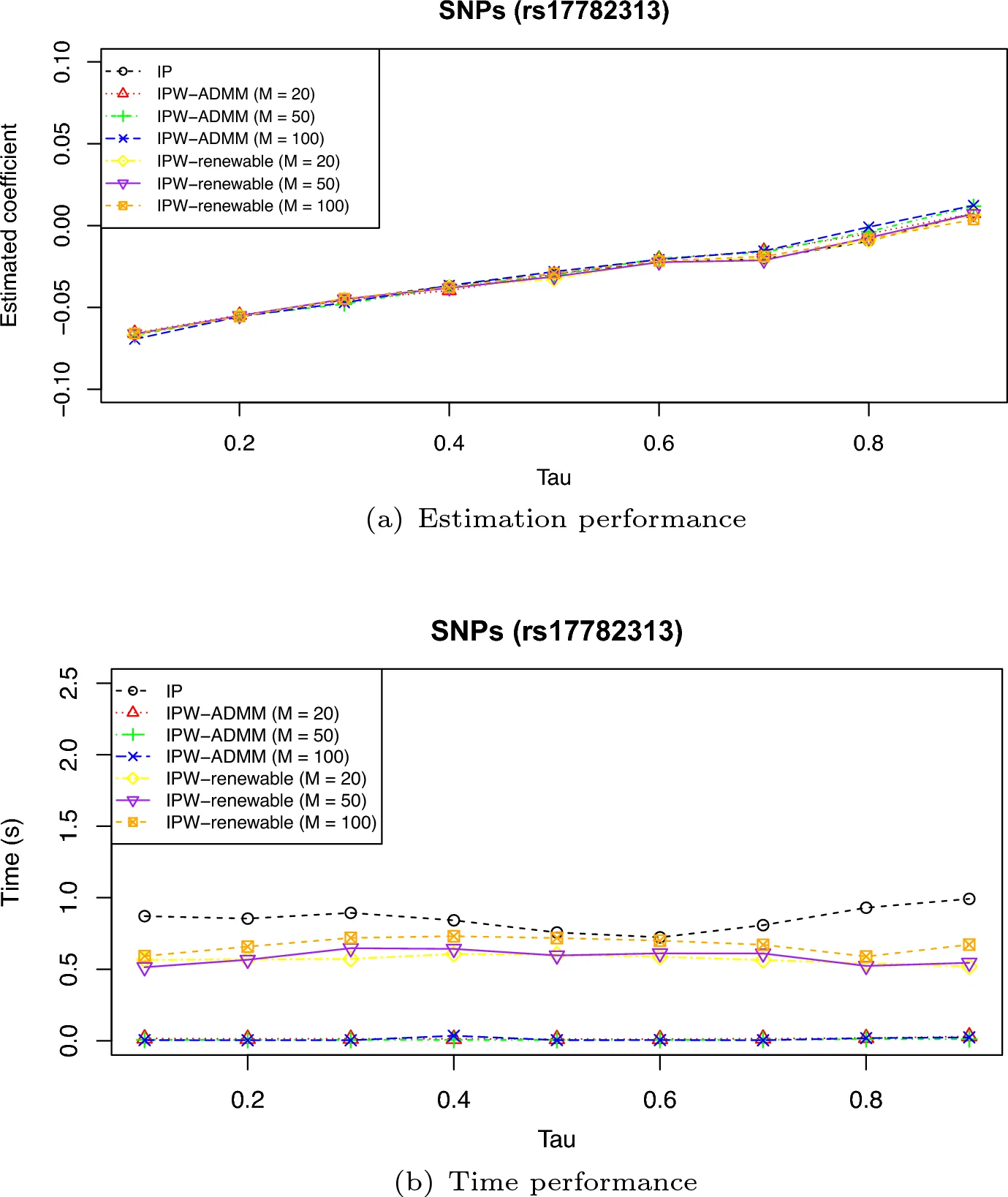
Comparison of IP, IPW-ADMM and IPW-renewable for UK Biobank data analysis

To emulate distributed computing, we partition the full dataset into M=20,50, and 100 equal-sized blocks (with the last block slightly larger when *n*/*M* is not an integer). These blocks are treated as independent data holders for evaluating the communication and computation efficiency of the two proposed algorithms. We compute the WQR estimates of the SNP’s effect at quantile levels $\tau = 0.1, 0.2, \ldots , 0.9$ using IPW-ADMM, IPW-renewable, and the oracle centralized analysis using the IP method. Figure 1a plots the WQR estimates across quantiles. The SNP exhibits a strong increasing effect across quantiles, with larger positive effects at higher BMI levels. All three methods produce nearly identical coefficient curves, with differences visually imperceptible.

Figure 1b reports the computation time for the three methods across quantiles and across different choices of partition numbers M=20,50,100. Several clear patterns emerge. IPW-ADMM is consistently the fastest method, with computation time effectively flat as the number of partitions *M* increases. This behavior reflects the full parallelizability of ADMM: more partitions reduce the per-block data size, so each local update becomes cheaper without introducing additional communication overhead. Second, IPW-renewable is slower overall because it processes blocks sequentially, but its runtime still remains substantially below that of the centralized IP method. For IPW-renewable, increasing *M* yields mild improvements in speed as the local block sizes shrink, though the gains are less pronounced than for ADMM because the total number of sequential updates also increases with *M*. Finally, the centralized IP method shows the highest and least variable runtime across quantiles, as it requires solving a full interior point problem on the complete dataset at each quantile.

Overall, this example illustrates that the proposed distributed algorithms maintain high statistical fidelity while achieving substantial computational advantages in a large-scale real data setting.

## Conclusion

This work addresses the challenge of performing QR in large-scale distributed settings with missing data. We proposed two two-stage algorithms, IPW-ADMM and IPW-renewable, that combine distributed estimation of inverse probability weights with scalable optimization for the WQR objective. IPW-ADMM employs a multi-block ADMM formulation that enables fully parallel updates, making it well suited for batch-distributed environments in which data are stored across multiple machines and global synchronization is feasible. In contrast, IPW-renewable provides a sequential alternative based on renewable summaries and smoothed scores and is particularly suitable when data arrive or are incorporated incrementally, or when storage or communication resources favor one-pass updating.

Our simulation studies showed that both estimators attain accuracy comparable to the centralized IP method while offering substantial reductions in computation time in distributed settings. The UK Biobank analysis further illustrated these advantages: all three estimators yield nearly identical coefficient estimates, with IPW-ADMM achieving the greatest computational time gains due to its parallel structure.

There remain several natural directions in which the proposed framework could be further extended. For example, although our methods are designed for MAR-type missingness with a correctly specified missingness model, our additional sensitivity analysis suggests that the distributed procedures can still track the centralized IPW benchmark closely under mild misspecification. A broader theoretical and empirical investigation of robustness to more substantial misspecification or to non-MAR mechanisms would be valuable in future work. Similarly, while the renewable estimator performs well empirically, a deeper theoretical investigation of its approximation properties could provide additional insight. It is also important to adapt the algorithms to accommodate communication constraints or heterogeneity across computing nodes, which would broaden their applicability to more complex distributed systems.

Overall, IPW-ADMM and IPW-renewable offer practical, accurate, and scalable tools for quantile regression with missing covariates in large distributed datasets. Their strong empirical performance and flexibility suggest broad potential in biomedical, health, and other data-intensive research domains.

## Supplementary Information

Below is the link to the electronic supplementary material.

Supplementary file 1 (pdf 184 KB)
