# Supplementary material for: Distributed quantile regression for big data with missing covariates
Source: Stat Biosci. 2026 Jul 9;18(3):844–75. doi: 10.1007/s12561-026-09528-6 (PMC13588827; doi:10.1007/s12561-026-09528-6)
Supplement: Supplementary file 1 [file 12561_2026_9528_MOESM1_ESM.pdf]

Supplementary Materials for "Distributed quantile regression for big data with missing covariates"

Ye Fan[1], Hua Zhou[2,3], Jin Zhou[3,4,5], Tiejun Tong[6], Nan Lin[7*]

[1]School of Statistics and Data Science, Capital University of Economics and Business, Beijing, 100070, China.
[2]Department of Computational Medicine, University of California, Los Angeles, CA 90095, USA.
[3]Department of Biostatistics, University of California, Los Angeles, CA 90095, USA.
[4]Department of Medicine, University of California, Los Angeles, CA 90095, USA.
[5]Department of Epidemiology and Biostatistics, University of Arizona, Tucson, AZ 85721, USA.
[6]Department of Mathematics, Hong Kong Baptist University, Hong Kong, 999077, China.
[7*]Department of Statistics and Data Science, Washington University in St. Louis, St. Louis, MO 63130, USA.

*Corresponding author(s). E-mail(s): nlin@wustl.edu;

In this file, we give the technical proofs for Theorems 1 and 2.

1 Proof of Theorem 1

Denote $\boldsymbol{e}_{m1}^{k}=\boldsymbol{\beta}_{m}^{k}-\boldsymbol{\beta}^{k}$, $\boldsymbol{e}_{m2}^{k}=\boldsymbol{y}_{m}^{*}-X_{m}^{*}\boldsymbol{\beta}_{m}^{k}-\boldsymbol{r}_{m}^{k}$ $(m=1,2,\cdots,M)$ and

$$D^{k}=\sum_{m=1}^{M}\left[\eta^{-1}\left\|\boldsymbol{u}_{m}^{k}-\boldsymbol{u}_{m}^{*}\right\|_{2}^{2}+\eta^{-1}\left\|\boldsymbol{v}_{m}^{k}-\boldsymbol{v}_{m}^{*}\right\|_{2}^{2}+\eta\left\|\boldsymbol{\beta}_{m}^{k}-\boldsymbol{\beta}_{m}^{*}\right\|_{2}^{2}+\eta\left\|X_{m}^{*}\left(\boldsymbol{\beta}_{m}^{k}-\boldsymbol{\beta}_{m}^{*}\right)\right\|_{2}^{2}\right].$$

Next, we prove that the sequence $\left\{D^{k},k=1,2,\cdots\right\}$ is decreasing.

Firstly, according to the assumption imposed in Theorem 1, $(\boldsymbol{\beta}^{*},\boldsymbol{r}_{m}^{*},\boldsymbol{\beta}_{m}^{*},\boldsymbol{u}_{m}^{*},\boldsymbol{v}_{m}^{*})$ is a saddle point of $\mathcal{L}_{0}\left(\boldsymbol{\beta},\boldsymbol{r}_{m},\boldsymbol{\beta}_{m},\boldsymbol{u}_{m},\boldsymbol{v}_{m}\right)$, and the following inequality holds at any iteration k,

$$\mathcal{L}_{0}\left(\boldsymbol{\beta}^{*},\boldsymbol{r}_{m}^{*},\boldsymbol{\beta}_{m}^{*},\boldsymbol{u}_{m}^{*},\boldsymbol{v}_{m}^{*}\right)\leq\mathcal{L}_{0}\left(\boldsymbol{\beta}^{k+1},\boldsymbol{r}_{m}^{k+1},\boldsymbol{\beta}_{m}^{k+1},\boldsymbol{u}_{m}^{*},\boldsymbol{v}_{m}^{*}\right).$$

By using the facts that $\boldsymbol{\beta}_{m}^{*}=\boldsymbol{\beta}^{*}$ and $\boldsymbol{r}_{m}^{*}=\boldsymbol{y}_{m}^{*}-X_{m}^{*}\boldsymbol{\beta}_{m}^{*}$, this inequality can be rewritten as

$$\sum_{m=1}^{M}\rho_{\tau}\left(\boldsymbol{r}_{m}^{*}\right)\leq\sum_{m=1}^{M}\rho_{\tau}\left(\boldsymbol{r}_{m}^{k+1}\right)+\sum_{m=1}^{M}\boldsymbol{u}_{m}^{*\mathrm{T}}\boldsymbol{e}_{m1}^{k+1}+\sum_{m=1}^{M}\boldsymbol{v}_{m}^{*\mathrm{T}}\boldsymbol{e}_{m2}^{k+1}. \tag{1}$$

Secondly, from the optimality of $\boldsymbol{\beta}^{k+1}$ and $\boldsymbol{\beta}_{m}^{k+1}$ $(m=1,2,\cdots,M)$ (see the subproblems (11) and (13) in the main article), we can get

$$\sum_{m=1}^{M}\left[\boldsymbol{u}_{m}^{k}+\eta\left(\boldsymbol{\beta}_{m}^{k}-\boldsymbol{\beta}^{k+1}\right)\right]=\mathbf{0}_{p}, \tag{2}$$

and

$$\boldsymbol{u}_{m}^{k}+\eta\left(\boldsymbol{\beta}_{m}^{k+1}-\boldsymbol{\beta}^{k+1}\right)-X_{m}^{*\mathrm{T}}\boldsymbol{v}_{m}^{k}-\eta X_{m}^{*\mathrm{T}}\left(\boldsymbol{y}_{m}^{*}-X_{m}^{*}\boldsymbol{\beta}_{m}^{k+1}-\boldsymbol{r}_{m}^{k+1}\right)=\mathbf{0}_{p}. \tag{3}$$

By substituting the equations (14)-(15) in the main article into (2)-(3), we obtain

$$\sum_{m=1}^{M}\left[\boldsymbol{u}_m^{k+1}-\eta\left(\boldsymbol{\beta}_m^{k+1}-\boldsymbol{\beta}_m^{k}\right)\right]=\mathbf{0}_p \quad \text{and} \quad \boldsymbol{u}_m^{k+1}-\boldsymbol{X}_m^{w\mathrm{T}}\boldsymbol{v}_m^{k+1}=\mathbf{0}_p. \tag{4}$$

Besides, by the convexity of the check loss function $\rho_\tau(\cdot)$ and the optimality of $\boldsymbol{r}_m^{k+1}$, we have

$$\mathbf{0}_{n_m^*}\in\partial\rho_\tau\left(\boldsymbol{r}_m^{k+1}\right)-\boldsymbol{v}_m^{k}-\eta\left(\boldsymbol{y}_m^*-X_m^*\boldsymbol{\beta}_m^k-\boldsymbol{r}_m^{k+1}\right). \tag{5}$$

From (5), we can see that optimizing the subproblem (12) in the main article is equivalent to solving

$$\boldsymbol{r}_m^{k+1}=\arg\min_{\boldsymbol{r}_m}\left\{\rho_\tau\left(\boldsymbol{r}_m\right)-\left[\boldsymbol{v}_m^{k+1}+\eta X_m^*\left(\boldsymbol{\beta}_m^{k+1}-\boldsymbol{\beta}_m^{k}\right)\right]^{\mathrm{T}}\boldsymbol{r}_m\right\}.$$

Then we have

$$\begin{aligned}&\sum_{m=1}^{M}\rho_\tau\left(\boldsymbol{r}_m^{k+1}\right)-\sum_{m=1}^{M}\left[\boldsymbol{v}_m^{k+1}+\eta X_m^*\left(\boldsymbol{\beta}_m^{k+1}-\boldsymbol{\beta}_m^{k}\right)\right]^{\mathrm{T}}\boldsymbol{r}_m^{k+1}\\ \leq&\sum_{m=1}^{M}\rho_\tau\left(\boldsymbol{r}_m^{*}\right)-\sum_{m=1}^{M}\left[\boldsymbol{v}_m^{k+1}+\eta X_m^*\left(\boldsymbol{\beta}_m^{k+1}-\boldsymbol{\beta}_m^{k}\right)\right]^{\mathrm{T}}\boldsymbol{r}_m^{*}.\end{aligned} \tag{6}$$

Multiplying the two equations in (4) by $\left(\boldsymbol{\beta}^{k+1}-\boldsymbol{\beta}^*\right)$ and $\left(\boldsymbol{\beta}_m^{k+1}-\boldsymbol{\beta}_m^*\right)$, respectively, we can get

$$\sum_{m=1}^{M}\left[\boldsymbol{u}_m^{k+1}-\eta\left(\boldsymbol{\beta}_m^{k+1}-\boldsymbol{\beta}_m^{k}\right)\right]^{\mathrm{T}}\left(\boldsymbol{\beta}^{k+1}-\boldsymbol{\beta}^*\right)=0, \tag{7}$$

and

$$\sum_{m=1}^{M}\left(\boldsymbol{u}_m^{k+1}-X_m^{*\mathrm{T}}\boldsymbol{v}_m^{k+1}\right)^{\mathrm{T}}\left(\boldsymbol{\beta}_m^{k+1}-\boldsymbol{\beta}_m^*\right)=0. \tag{8}$$

Through adding (7) and (8) to the inequality (6), we obtain

$$
\begin{aligned}
\sum_{m=1}^{M} \rho_\tau\left(\boldsymbol{r}_m^{k+1}\right) \leq & \sum_{m=1}^{M} \rho_\tau\left(\boldsymbol{r}_m^{*}\right) - \sum_{m=1}^{M}\left[\boldsymbol{v}_m^{k+1} + \eta X_m^{*}\left(\boldsymbol{\beta}_m^{k+1} - \boldsymbol{\beta}_m^{k}\right)\right]^{\mathrm{T}} \boldsymbol{r}_m^{*} \\
& + \sum_{m=1}^{M}\left[\boldsymbol{v}_m^{k+1} + \eta X_m^{*}\left(\boldsymbol{\beta}_m^{k+1} - \boldsymbol{\beta}_m^{k}\right)\right]^{\mathrm{T}} \boldsymbol{r}_m^{k+1} \\
& + \sum_{m=1}^{M}\left[\boldsymbol{u}_m^{k+1} - \eta\left(\boldsymbol{\beta}_m^{k+1} - \boldsymbol{\beta}_m^{k}\right)\right]^{\mathrm{T}}\left(\boldsymbol{\beta}^{k+1} - \boldsymbol{\beta}^{*}\right) \\
& - \sum_{m=1}^{M}\left(\boldsymbol{u}_m^{k+1} - X_m^{*\mathrm{T}} \boldsymbol{v}_m^{k+1}\right)^{\mathrm{T}}\left(\boldsymbol{\beta}_m^{k+1} - \boldsymbol{\beta}_m^{*}\right).
\end{aligned} \tag{9}
$$

Rearranging (9), we have

$$
\begin{aligned}
\sum_{m=1}^{M} \rho_\tau\left(\boldsymbol{r}_m^{k+1}\right) \leq & \sum_{m=1}^{M} \rho_\tau\left(\boldsymbol{r}_m^{*}\right) - \sum_{m=1}^{M}\left(\boldsymbol{u}_m^{k+1}\right)^{\mathrm{T}}\left[\left(\boldsymbol{\beta}_m^{k+1} - \boldsymbol{\beta}^{k+1}\right) - \left(\boldsymbol{\beta}_m^{*} - \boldsymbol{\beta}^{*}\right)\right] \\
& - \sum_{m=1}^{M}\left(\boldsymbol{v}_m^{k+1}\right)^{\mathrm{T}}\left[\left(X_m^{*}\boldsymbol{\beta}_m^{*} + \boldsymbol{r}_m^{*}\right) - \left(X_m^{*}\boldsymbol{\beta}_m^{k+1} + \boldsymbol{r}_m^{k+1}\right)\right] \\
& + \sum_{m=1}^{M} \eta\left[X_m^{*}\left(\boldsymbol{\beta}_m^{k+1} - \boldsymbol{\beta}_m^{k}\right)\right]^{\mathrm{T}}\left(\boldsymbol{r}_m^{k+1} - \boldsymbol{r}_m^{*}\right) \\
& - \sum_{m=1}^{M} \eta\left(\boldsymbol{\beta}_m^{k+1} - \boldsymbol{\beta}_m^{k}\right)^{\mathrm{T}}\left(\boldsymbol{\beta}^{k+1} - \boldsymbol{\beta}^{*}\right).
\end{aligned} \tag{10}
$$

By applying the facts that $\boldsymbol{\beta}_m^{*} = \boldsymbol{\beta}^{*}$ and $\boldsymbol{r}_m^{*} = \boldsymbol{y}_m^{*} - X_m^{*}\boldsymbol{\beta}_m^{*}$, and the definitions of $\boldsymbol{e}_{m1}^{k+1}$ and $\boldsymbol{e}_{m2}^{k+1}$, we can rewrite (10) as

$$
\begin{aligned}
\sum_{m=1}^{M} \rho_\tau\left(\boldsymbol{r}_m^{k+1}\right) \leq & \sum_{m=1}^{M} \rho_\tau\left(\boldsymbol{r}_m^{*}\right) - \sum_{m=1}^{M}\left(\boldsymbol{u}_m^{k+1}\right)^{\mathrm{T}} \boldsymbol{e}_{m1}^{k+1} - \sum_{m=1}^{M}\left(\boldsymbol{v}_m^{k+1}\right)^{\mathrm{T}} \boldsymbol{e}_{m2}^{k+1} \\
& + \sum_{m=1}^{M} \eta\left[X_m^{*}\left(\boldsymbol{\beta}_m^{k+1} - \boldsymbol{\beta}_m^{k}\right)\right]^{\mathrm{T}}\left(\boldsymbol{r}_m^{k+1} - \boldsymbol{r}_m^{*}\right) \\
& - \sum_{m=1}^{M} \eta\left(\boldsymbol{\beta}_m^{k+1} - \boldsymbol{\beta}_m^{k}\right)^{\mathrm{T}}\left(\boldsymbol{\beta}^{k+1} - \boldsymbol{\beta}^{*}\right).
\end{aligned} \tag{11}
$$

Besides, noting that $\left(\boldsymbol{r}_m^{k+1} - \boldsymbol{r}_m^*\right)$ and $\left(\boldsymbol{\beta}^{k+1} - \boldsymbol{\beta}^*\right)$ are respectively equal to $\left[-\boldsymbol{e}_{m2}^{k+1} - X_m^*\left(\boldsymbol{\beta}_m^{k+1} - \boldsymbol{\beta}_m^*\right)\right]$ and $\left[-\boldsymbol{e}_{m1}^{k+1} + \left(\boldsymbol{\beta}_m^{k+1} - \boldsymbol{\beta}_m^*\right)\right]$, the inequality (11) can be further rewritten as

$$\begin{aligned}\sum_{m=1}^{M} \rho_\tau\left(\boldsymbol{r}_m^{k+1}\right) \leq \sum_{m=1}^{M} \rho_\tau\left(\boldsymbol{r}_m^*\right) &- \sum_{m=1}^{M}\left(\boldsymbol{u}_m^{k+1}\right)^{\mathrm{T}} \boldsymbol{e}_{m1}^{k+1} - \sum_{m=1}^{M}\left(\boldsymbol{v}_m^{k+1}\right)^{\mathrm{T}} \boldsymbol{e}_{m2}^{k+1} \\ &+ \sum_{m=1}^{M} \eta\left[X_m^*\left(\boldsymbol{\beta}_m^{k+1} - \boldsymbol{\beta}_m^k\right)\right]^{\mathrm{T}}\left[-\boldsymbol{e}_{m2}^{k+1} - X_m^*\left(\boldsymbol{\beta}_m^{k+1} - \boldsymbol{\beta}_m^*\right)\right] \\ &+ \sum_{m=1}^{M} \eta\left(\boldsymbol{\beta}_m^{k+1} - \boldsymbol{\beta}_m^k\right)^{\mathrm{T}}\left[\boldsymbol{e}_{m1}^{k+1} - \left(\boldsymbol{\beta}_m^{k+1} - \boldsymbol{\beta}_m^*\right)\right].\end{aligned} \tag{12}$$

Combining (1) and (12), we get

$$\begin{aligned}&\sum_{m=1}^{M}\left(\boldsymbol{u}_m^{k+1} - \boldsymbol{u}_m^*\right)^{\mathrm{T}} \boldsymbol{e}_{m1}^{k+1} + \sum_{m=1}^{M}\left(\boldsymbol{v}_m^{k+1} - \boldsymbol{v}_m^*\right)^{\mathrm{T}} \boldsymbol{e}_{m2}^{k+1} \\ \leq &\sum_{m=1}^{M} \eta\left[X_m^*\left(\boldsymbol{\beta}_m^{k+1} - \boldsymbol{\beta}_m^k\right)\right]^{\mathrm{T}}\left[-\boldsymbol{e}_{m2}^{k+1} - X_m^*\left(\boldsymbol{\beta}_m^{k+1} - \boldsymbol{\beta}_m^*\right)\right] \\ + &\sum_{m=1}^{M} \eta\left(\boldsymbol{\beta}_m^{k+1} - \boldsymbol{\beta}_m^k\right)^{\mathrm{T}}\left[\boldsymbol{e}_{m1}^{k+1} - \left(\boldsymbol{\beta}_m^{k+1} - \boldsymbol{\beta}_m^*\right)\right].\end{aligned} \tag{13}$$

On the other hand, by the definition of D^k, we have

$$\begin{aligned}D^{k+1} - D^k = &-\eta \sum_{m=1}^{M}\left\|\boldsymbol{e}_{m1}^{k+1}\right\|_2^2 + 2\sum_{m=1}^{M}\left(\boldsymbol{u}_m^{k+1} - \boldsymbol{u}_m^*\right)^{\mathrm{T}} \boldsymbol{e}_{m1}^{k+1} \\ &-\eta \sum_{m=1}^{M}\left\|\boldsymbol{e}_{m2}^{k+1}\right\|_2^2 + 2\sum_{m=1}^{M}\left(\boldsymbol{v}_m^{k+1} - \boldsymbol{v}_m^*\right)^{\mathrm{T}} \boldsymbol{e}_{m2}^{k+1} \\ &-\eta \sum_{m=1}^{M}\left\|\boldsymbol{\beta}_m^{k+1} - \boldsymbol{\beta}_m^k\right\|_2^2 + 2\sum_{m=1}^{M}\left(\boldsymbol{\beta}_m^{k+1} - \boldsymbol{\beta}_m^k\right)^{\mathrm{T}}\left(\boldsymbol{\beta}_m^{k+1} - \boldsymbol{\beta}_m^*\right) \\ &-\eta \sum_{m=1}^{M}\left\|X_m^*\left(\boldsymbol{\beta}_m^{k+1} - \boldsymbol{\beta}_m^k\right)\right\|_2^2 + 2\sum_{m=1}^{M}\left[X_m^*\left(\boldsymbol{\beta}_m^{k+1} - \boldsymbol{\beta}_m^k\right)\right]^{\mathrm{T}}\left[X_m^*\left(\boldsymbol{\beta}_m^{k+1} - \boldsymbol{\beta}_m^*\right)\right].\end{aligned}$$

By substituting (13) into the above equation, we get

$$
\begin{aligned}
D^{k+1} - D^{k} \leq & -\eta \sum_{m=1}^{M} \left[\|\boldsymbol{e}_{m1}^{k+1}\|_2^2 + \|\boldsymbol{e}_{m2}^{k+1}\|_2^2 + \|\boldsymbol{\beta}_m^{k+1} - \boldsymbol{\beta}_m^{k}\|_2^2 + \|X_m^* \left(\boldsymbol{\beta}_m^{k+1} - \boldsymbol{\beta}_m^{k}\right)\|_2^2 \right] \\
& + \sum_{m=1}^{M} \eta \left(\boldsymbol{\beta}_m^{k+1} - \boldsymbol{\beta}_m^{k}\right)^{\mathrm{T}} \boldsymbol{e}_{m1}^{k+1} - \sum_{m=1}^{M} \eta \left[X_m^* \left(\boldsymbol{\beta}_m^{k+1} - \boldsymbol{\beta}_m^{k}\right)\right]^{\mathrm{T}} \boldsymbol{e}_{m2}^{k+1} \\
\leq & -\eta \sum_{m=1}^{M} \left[\|\boldsymbol{e}_{m1}^{k+1}\|_2^2 + \|\boldsymbol{e}_{m2}^{k+1}\|_2^2 + \|\boldsymbol{\beta}_m^{k+1} - \boldsymbol{\beta}_m^{k}\|_2^2 + \|X_m^* \left(\boldsymbol{\beta}_m^{k+1} - \boldsymbol{\beta}_m^{k}\right)\|_2^2 \right],
\end{aligned} \tag{14}
$$

where the second inequality is obtained by using (4) and the fact that

$$
\begin{aligned}
& \sum_{m=1}^{M} \eta \left(\boldsymbol{\beta}_m^{k+1} - \boldsymbol{\beta}_m^{k}\right)^{\mathrm{T}} \boldsymbol{e}_{m1}^{k+1} - \sum_{m=1}^{M} \eta \left[X_m^* \left(\boldsymbol{\beta}_m^{k+1} - \boldsymbol{\beta}_m^{k}\right)\right]^{\mathrm{T}} \boldsymbol{e}_{m2}^{k+1} \\
= & \sum_{m=1}^{M} \left(\boldsymbol{\beta}_m^{k+1} - \boldsymbol{\beta}_m^{k}\right)^{\mathrm{T}} \left[\left(\boldsymbol{u}_m^{k+1} - X_m^{*\mathrm{T}} \boldsymbol{v}_m^{k+1}\right) - \left(\boldsymbol{u}_m^{k} - X_m^{*\mathrm{T}} \boldsymbol{v}_m^{k}\right) \right] = 0.
\end{aligned}
$$

The inequality (14) implies that D^k is decreasing with respect to k. Meanwhile, we have $D^k \geq 0$ by its definition. Therefore, the sequence $\left\{D^k, k = 1, 2, \cdots\right\}$ must be convergent and hence we have $\boldsymbol{e}_{m1}^{k+1} = \boldsymbol{\beta}_m^{k+1} - \boldsymbol{\beta}^{k+1} \to \mathbf{0}_p$, $\boldsymbol{e}_{m2}^{k+1} = \boldsymbol{y}_m^* - X_m^* \boldsymbol{\beta}_m^{k+1} - \boldsymbol{r}_m^{k+1} \to \mathbf{0}_{n_m^*}$, $\boldsymbol{\beta}_m^{k+1} \to \boldsymbol{\beta}_m^{k}$ and $X_m^* \boldsymbol{\beta}_m^{k+1} \to X_m^* \boldsymbol{\beta}_m^{k}$. That is to say, there must exist $\tilde{\boldsymbol{\beta}}_m^*$, $\tilde{\boldsymbol{\beta}}^*$ and $\tilde{\boldsymbol{r}}_m^*$, such that $\boldsymbol{\beta}_m^{k+1} \to \tilde{\boldsymbol{\beta}}_m^*$, $X_m^* \boldsymbol{\beta}_m^{k+1} \to X_m^* \tilde{\boldsymbol{\beta}}_m^*$, $\boldsymbol{\beta}^{k+1} \to \tilde{\boldsymbol{\beta}}^*$ and $\boldsymbol{r}_m^{k+1} \to \tilde{\boldsymbol{r}}_m^*$. In addition, $\tilde{\boldsymbol{\beta}}_m^* - \tilde{\boldsymbol{\beta}}^* = \mathbf{0}_p$ and $\boldsymbol{y}_m^* - X_m^* \tilde{\boldsymbol{\beta}}_m^* - \tilde{\boldsymbol{r}}_m^* = \mathbf{0}_{n_m^*}$ hold, which means $\left(\tilde{\boldsymbol{\beta}}^*, \tilde{\boldsymbol{r}}_m^*, \tilde{\boldsymbol{\beta}}_m^*\right)$ is a feasible solution to the problem (10) in the main article. Besides, from the fact that $D^{k+1} \leq D^0$, we know that both $\boldsymbol{\beta}_m^{k+1}$ and $X_m^* \boldsymbol{\beta}_m^{k+1}$ are bounded. Then from the inequality (12), we finally obtain

$$
\lim_{k \to \infty} \sum_{m=1}^{M} \rho_\tau\left(\boldsymbol{r}_m^{k+1}\right) = \sum_{m=1}^{M} \rho_\tau\left(\tilde{\boldsymbol{r}}_m^*\right) = \sum_{m=1}^{M} \rho_\tau\left(\boldsymbol{r}_m^*\right),
$$

and hence $\left(\tilde{\boldsymbol{\beta}}^{*}, \tilde{\boldsymbol{r}}_{m}^{*}, \tilde{\boldsymbol{\beta}}_{m}^{*}\right)$ is actually an optimal solution to (10) in the main article.

2 Proof of Theorem 2

2.1 Proof for the IPW-renewable estimator's consistency

We prove this consistency by using the induction technique. In the implementation of IPW-renewable, if a consistent initial estimator is not available, we set the initial value of the algorithm as the solution to $Q_{n_1^*}(\boldsymbol{\beta}) = \mathbf{0}_p$, from which the obtained estimator $\hat{\boldsymbol{\beta}}^{(1)}$ is $\sqrt{n_1^*}$-consistent for $\boldsymbol{\beta}_0$ (He et al. 2023), as this local estimation process does not involve any approximation. Next, we assume that the estimators $\hat{\boldsymbol{\beta}}^{(m)}(m = 1, 2, \cdots, M-1, M \geq 1)$ are consistent, and prove that $\hat{\boldsymbol{\beta}}^{(M)}$ is also consistent.

From the algorithm details of IPW-renewable given in Algorithm 2 in the main article, we know that $\hat{\boldsymbol{\beta}}^{(M)}$ is obtained by solving

$$Q_{n_M^*}(\boldsymbol{\beta}) + \sum_{m=1}^{M-1} \nabla Q_{n_m^*}\left(\hat{\boldsymbol{\beta}}^{(m)}\right)\left(\boldsymbol{\beta} - \hat{\boldsymbol{\beta}}^{(M-1)}\right) = \mathbf{0}_p.$$

That is, we have

$$Q_{n_M^*}\left(\hat{\boldsymbol{\beta}}^{(M)}\right) + \sum_{m=1}^{M-1} \nabla Q_{n_m^*}\left(\hat{\boldsymbol{\beta}}^{(m)}\right)\left(\hat{\boldsymbol{\beta}}^{(M)} - \hat{\boldsymbol{\beta}}^{(M-1)}\right) = \mathbf{0}_p. \tag{15}$$

On the other hand, by utilizing the fact that $E\left[Q\left(\boldsymbol{\beta}_0\right)\right] = \mathbf{0}_p$, the Law of Large Numbers and Assumption 1(c), which implies that $n_1^* \to \infty$ and further $n^* = \sum_{m=1}^{M} n_m^* \to \infty$ under our setup of the initial value for the algorithm, we can obtain

$$\frac{1}{n^*} Q_{n_M^*}\left(\boldsymbol{\beta}_0\right) + \frac{1}{n^*}\left[-\sum_{m=1}^{M-1} \nabla Q_{n_m^*}\left(\hat{\boldsymbol{\beta}}^{(m)}\right)\right]\left(\hat{\boldsymbol{\beta}}^{(M-1)} - \boldsymbol{\beta}_0\right) = o_p(1). \tag{16}$$

Combining (15) and (16), we then have

$$\frac{1}{n^*}\left[Q_{n_M^*}\left(\boldsymbol{\beta}_0\right)-Q_{n_M^*}\left(\hat{\boldsymbol{\beta}}^{(M)}\right)\right]+\frac{1}{n^*}\left[-\sum_{m=1}^{M-1}\nabla Q_{n_m^*}\left(\hat{\boldsymbol{\beta}}^{(m)}\right)\right]\left(\hat{\boldsymbol{\beta}}^{(M)}-\boldsymbol{\beta}_0\right)=o_p\left(1\right). \tag{17}$$

Besides, by taking the first-order Taylor expansion for $Q_{n_M^*}\left(\hat{\boldsymbol{\beta}}^{(M)}\right)$ at $\boldsymbol{\beta}_0$, we have

$$Q_{n_M^*}\left(\hat{\boldsymbol{\beta}}^{(M)}\right)-Q_{n_M^*}\left(\boldsymbol{\beta}_0\right)=\left[\nabla Q_{n_M^*}\left(\boldsymbol{\xi}_M\right)-\nabla Q_{n_M^*}\left(\boldsymbol{\beta}_0\right)+\nabla Q_{n_M^*}\left(\boldsymbol{\beta}_0\right)\right]\left(\hat{\boldsymbol{\beta}}^{(M)}-\boldsymbol{\beta}_0\right),$$

where

$$\left\|\nabla Q_{n_M^*}\left(\boldsymbol{\xi}_M\right)-\nabla Q_{n_M^*}\left(\boldsymbol{\beta}_0\right)\right\|_2\leq C_M\left\|\boldsymbol{\xi}_M-\boldsymbol{\beta}_0\right\|_2\leq C_M\left\|\hat{\boldsymbol{\beta}}^{(M)}-\boldsymbol{\beta}_0\right\|_2,$$

under Assumption 1(b), which ensures the Lipschitz continuity of the Hessian matrix $\nabla Q_{n_M^*}\left(\boldsymbol{\beta}\right)$. Here $\boldsymbol{\xi}_M$ lies between $\hat{\boldsymbol{\beta}}^{(M)}$ and $\boldsymbol{\beta}_0$, and C_M is a constant bounded by the local sample size n_M^*. Then we have

$$Q_{n_M^*}\left(\hat{\boldsymbol{\beta}}^{(M)}\right)-Q_{n_M^*}\left(\boldsymbol{\beta}_0\right)=\nabla Q_{n_M^*}\left(\boldsymbol{\beta}_0\right)\left(\hat{\boldsymbol{\beta}}^{(M)}-\boldsymbol{\beta}_0\right)+O_p\left(n_M^*\left\|\hat{\boldsymbol{\beta}}^{(M)}-\boldsymbol{\beta}_0\right\|_2^2\right). \tag{18}$$

Combining (17) and (18), we obtain

$$\frac{1}{n^*}\left[-\sum_{m=1}^{M-1}\nabla Q_{n_m^*}\left(\hat{\boldsymbol{\beta}}^{(m)}\right)-\nabla Q_{n_M^*}\left(\boldsymbol{\beta}_0\right)\right]\left(\hat{\boldsymbol{\beta}}^{(M)}-\boldsymbol{\beta}_0\right)+O_p\left(\frac{n_M^*}{n^*}\left\|\hat{\boldsymbol{\beta}}^{(M)}-\boldsymbol{\beta}_0\right\|_2^2\right)=o_p(1), \tag{19}$$

which then implies $\left\|\hat{\boldsymbol{\beta}}^{(M)}-\boldsymbol{\beta}_0\right\|_2=o_p(1)$. This completes the proof for the consistency.

2.2 Proof for the IPW-renewable estimator's asymptotic normality

Firstly, by taking the first-order Taylor expansion for $Q_{n_1^*}(\boldsymbol{\beta}_0)$ at $\hat{\boldsymbol{\beta}}^{(1)}$, we have

$$Q_{n_1^*}(\boldsymbol{\beta}_0) = -\nabla Q_{n_1^*}\left(\hat{\boldsymbol{\beta}}^{(1)}\right)\left(\hat{\boldsymbol{\beta}}^{(1)} - \boldsymbol{\beta}_0\right) + O_p\left(n_1^*\left\|\hat{\boldsymbol{\beta}}^{(1)} - \boldsymbol{\beta}_0\right\|_2^2\right),$$

where we utilized the fact that $Q_{n_1^*}\left(\hat{\boldsymbol{\beta}}^{(1)}\right) = \mathbf{0}_p$. Besides, by combining (15) and (18), we have

$$\begin{aligned} Q_{n_m^*}(\boldsymbol{\theta}_0) = &\left[-\sum_{j=1}^{m-1}\nabla Q_{n_j^*}\left(\hat{\boldsymbol{\beta}}^{(j)}\right) - \nabla Q_{n_m^*}(\boldsymbol{\beta}_0)\right]\left(\hat{\boldsymbol{\beta}}^{(m)} - \boldsymbol{\beta}_0\right) \\ &+ \left[\sum_{j=1}^{m-1}\nabla Q_{n_j^*}\left(\hat{\boldsymbol{\beta}}^{(j)}\right)\right]\left(\hat{\boldsymbol{\beta}}^{(m-1)} - \boldsymbol{\beta}_0\right) + O_p\left(n_m^*\left\|\hat{\boldsymbol{\beta}}^{(m)} - \boldsymbol{\beta}_0\right\|\right), \end{aligned}$$

holding for $m \geq 2$. Cumulating $Q_{n_m^*}(\boldsymbol{\theta}_0)$'s by m, we then obtain

$$\begin{aligned} \sum_{m=1}^{M} Q_{n_m^*}(\boldsymbol{\theta}_0) &= \left[-\sum_{m=1}^{M-1}\nabla Q_{n_m^*}\left(\hat{\boldsymbol{\beta}}^{(M)}\right) - \nabla Q_{n_M^*}(\boldsymbol{\beta}_0)\right]\left(\hat{\boldsymbol{\beta}}^{(M)} - \boldsymbol{\beta}_0\right) + O_p\left(\sum_{m=1}^{M} n_m^*\left\|\hat{\boldsymbol{\beta}}^{(m)} - \boldsymbol{\beta}_0\right\|_2^2\right) \\ &= \left[-\sum_{m=1}^{M}\nabla Q_{n_m^*}(\boldsymbol{\beta}_0)\right]\left(\hat{\boldsymbol{\beta}}^{(M)} - \boldsymbol{\beta}_0\right) + O_p\left(\sum_{m=1}^{M} n_m^*\left\|\hat{\boldsymbol{\beta}}^{(m)} - \boldsymbol{\beta}_0\right\|_2^2\right), \end{aligned} \tag{20}$$

and further

$$\frac{1}{n^*}\sum_{m=1}^{M} Q_{n_m^*}(\boldsymbol{\beta}_0) = \frac{1}{n^*}\left[-\sum_{m=1}^{M}\nabla Q_{n_m^*}(\boldsymbol{\beta}_0)\right]\left(\hat{\boldsymbol{\beta}}^{(M)} - \boldsymbol{\beta}_0\right) + O_p\left(\frac{1}{n^*}\sum_{m=1}^{M} n_m^*\left\|\hat{\boldsymbol{\beta}}^{(m)} - \boldsymbol{\beta}_0\right\|_2^2\right). \tag{21}$$

Note that all the estimators $\hat{\boldsymbol{\beta}}^{(m)}$'s $(m \geq 1)$ are consistent, and thus (21) implies that the term $\frac{1}{n^*}\left[-\sum_{m=1}^{M} \nabla Q_{n_m^*}(\boldsymbol{\beta}_0)\right]\left(\hat{\boldsymbol{\beta}}^{(M)} - \boldsymbol{\beta}_0\right)$ is asymptotically equivalent to $\frac{1}{n^*}\sum_{m=1}^{M} Q_{n_m^*}(\boldsymbol{\beta}_0)$. On the other hand, by the Central Limit Theorem we have

$$\frac{1}{\sqrt{n^*}}\sum_{m=1}^{M} Q_{n_m^*}(\boldsymbol{\beta}_0) \rightarrow_d \mathcal{N}\left(\mathbf{0}_p, E\left(Q(\boldsymbol{\beta}_0)\, Q^{\mathrm{T}}(\boldsymbol{\beta}_0)\right)\right),$$

and by the Law of Large Numbers we have

$$\frac{1}{n^*}\left[\sum_{m=1}^{M} \nabla Q_{n_m^*}(\boldsymbol{\beta}_0)\right] \rightarrow \nabla Q(\boldsymbol{\beta}_0). \tag{22}$$

And the asymptotic normality follows.

References

He X, Pan X, Tan KM, et al (2023) Smoothed quantile regression with large-scale inference. Journal of Econometrics 232(2):367–388. https://doi.org/10.1016/j.jeconom.2021.07.010
